# Paired ATAC- and RNA-seq offer insight into the impact of HIV on alveolar macrophages: a pilot study

**DOI:** 10.1038/s41598-023-42644-7

**Published:** 2023-09-15

**Authors:** Bashar S. Staitieh, Xin Hu, Samantha M. Yeligar, Sara C. Auld

**Affiliations:** 1 Division of Pulmonary, Allergy, Critical Care, and Sleep Medicine, Department of Medicine, School of Medicine, Emory University, 615 Michael St NE, Ste 200, Atlanta, GA 30322 USA; 2 Grady Health System, Atlanta, GA USA; 3 Veterans Affairs Atlanta Healthcare System, Decatur, GA USA; 4 Gangarosa Department of Environmental Health, Rollins School of Public Health, Emory University, Atlanta, GA USA; 5 Departments of Epidemiology and Global Health, Rollins School of Public Health, Emory University, Atlanta, GA USA

**Keywords:** Applied immunology, Infectious diseases, Mucosal immunology, Translational research

## Abstract

People with HIV remain at greater risk for both infectious and non-infectious pulmonary diseases even after antiretroviral therapy initiation and CD4 cell count recovery. These clinical risks reflect persistent HIV-mediated defects in innate and adaptive immunity, including in the alveolar macrophage, a key innate immune effector in the lungs. In this proof-of-concept pilot study, we leveraged paired RNA-seq and ATAC-seq analyses of human alveolar macrophages obtained with research bronchoscopy from people with and without HIV to highlight the potential for recent methodologic advances to generate novel hypotheses about biological pathways that may contribute to impaired pulmonary immune function in people with HIV. In addition to 35 genes that were differentially expressed in macrophages from people with HIV, gene set enrichment analysis identified six gene sets that were differentially regulated. ATAC-seq analysis revealed 115 genes that were differentially accessible for people with HIV. Data-driven integration of the findings from these complementary, high-throughput techniques using xMWAS identified distinct clusters involving lipoprotein lipase and inflammatory pathways. By bringing together transcriptional and epigenetic data, this analytic approach points to several mechanisms, including previously unreported pathways, that warrant further exploration as potential mediators of the increased risk of pulmonary disease in people with HIV.

## Introduction

Despite increasing availability and uptake of antiretroviral therapy (ART), people with HIV remain at higher risk for both infectious and non-infectious pulmonary diseases. While rates of pneumonia have declined since the pre-ART era, pulmonary infections with both typical and atypical bacteria continue to be more common in people with HIV^1,2^. Rates of opportunistic infections with pathogens such as *Mycobacterium tuberculosis* (*Mtb*) and *Pneumocystis jirovecii* (*PCP*) remain similarly elevated^3–5^. At the same time, people with HIV are more likely to have impaired lung function and non-infectious chronic lung diseases such as emphysema^6–9^.

HIV-mediated defects in innate and adaptive immunity persist even after ART initiation and CD4 cell count recovery^10^. Pulmonary innate immunity is significantly impacted by the effects of HIV on alveolar macrophages. These defects, which include impairments in cellular redox balance, phagocytosis, caspase activation, and cytotoxic capacity, contribute to the continued risk for infectious pulmonary complications^11–13^. The lungs, and alveolar macrophages in particular, also serve as a reservoir for HIV and harbor residual virus, which, in turn, drives chronic immune activation and exhaustion^14,15^. This chronic immune activation then increases local levels of degradative and profibrotic mediators such as matrix metalloproteinases and TGF-β^16,17^, and likely underlies the increased risk for chronic lung disease in people with HIV.

Alveolar macrophages play a key role in the lungs, serving as both the first line of defense against pathogens in the lower airway and as mediators of tissue repair and recovery. HIV is known to alter macrophage function and phenotype, in addition to its effects of cell death pathways and pathogen killing^13,15,18^. However, despite these advances in our appreciation for the wide-ranging impacts of HIV infection on lung health, our understanding of the impacts of HIV on alveolar macrophage function remains limited. In this proof-of-concept study, we integrated RNA-seq and ATAC-seq analyses of alveolar macrophages from people with and without HIV to enable unbiased transcriptomic and epigenomic discovery of immune pathways altered by HIV infection.

## Methods

### Human subjects and ethics

From September 2018 to January 2019, study participants were prospectively recruited from primary care clinics affiliated with Grady Memorial Hospital in Atlanta, Georgia. All participants were at least 21 years of age with no history of either active TB disease, latent TB infection, prior treatment for either TB disease or infection, and a negative QuantiFERON-TB Gold test. Participants with HIV were on ART for at least 18 months with a CD4 cell count greater than 500 cells/µl and an undetectable HIV viral load. Exclusion criteria were as follows: pregnancy or breast feeding, receipt of immunosuppressive medications (≥ 15 mg per day of prednisone equivalent) within 30 days of screening, history of cirrhosis, cardiomyopathy, chronic kidney disease (≥ CKD3), known bleeding disorder or thrombocytopenia, or poorly controlled asthma or emphysema. Participants underwent flexible bronchoscopy with bronchoalveolar lavage (BAL) of a single lobe with sequential instillation of six 30 mL aliquots of normal saline solution. All participants provided written informed consent prior to any study procedures.

The study was approved by the Emory University Institutional Review Board and the Grady Memorial Hospital Research Oversight Committee (Emory IRB #00088993) and all research was performed in accordance with the relevant guidelines and regulations.

### Alveolar macrophage isolation, library preparation and sequencing

Alveolar macrophages were isolated from BAL of participants by centrifugation. Each sample was washed once in serum-free RPMI-1640 (ATCC) prior to plating in media with 10% FBS and gentamycin/amphotericin. After approximately two hours to allow the macrophages to adhere to the surface of the plate, the media was removed and refreshed to purify the macrophage population. Alveolar macrophage purity was determined to be ∼90–95% as measured by Diff-Quik (Dade Behring) staining and cell counting^19^. For RNA sample preparation, plated cells were lysed with RNA lysis buffer (Zymo Research, Irvine, CA) prior to sequencing on the Illumina NextSeq platform (high output, 150 cycles). ATAC-seq library preparation was undertaken using the procedure outlined by Buenrostro, et al^20^. Briefly, 50,000 cells were tagmented and fragment sizes were selected using magnetic beads. Library sequences were then amplified by PCR prior to post-PCR bead size selection to capture the appropriate tagmented DNA. After library quality control was confirmed, libraries were sequenced using the Ilumina NextSeq platform using primer sequences from Buenrostro et al.^21^.

### RNA-seq

Quality control and alignment of FASTQ files were performed using the standard workflow at Emory Integrated Computational Core^22^. Briefly, quality control was performed using the FastQC and sequences are aligned to the HG38 reference genome using STAR aligner^23,24^. The read counts were quantified using HTSeq-count and filtered to retain genes that have ≥ 10 for at least 30% of samples^23^. In total, 26,485 transcripts were detected and submitted for downstream statistical analysis.

The read counts were normalized by the variance stabilization transformation as implemented in DESeq2^25^. Principle component analysis and unsupervised hierarchical clustering were applied to assess sample distance and data from one HIV-negative participant were detected as an outlier (Supplemental Fig S1). This sample was therefore removed from the downstream RNA-seq and ATAC-seq statistical analysis. Differential expression gene analysis was performed using DESeq2^25^. Gene set enrichment analysis was further used to identify enrichment of functionally related genes in alveolar macrophages from people with HIV as compared to those without HIV^26^.

### ATAC-seq

Quality control and alignment of FASTQ files were performed using the standard workflow at Emory Integrated Computation Core^27^. Briefly, quality checks were performed on sequences using FastQC and processed with Trimmomatic^28^. Reads were aligned to HG38 with Burrows-Wheeler Aligner. The package SAMtools was used to remove duplicative, unmapped or non-uniquely mapped reads, and mitochondrial DNA reads^29^. Reads were adjusted for the 9 bp target sequence duplication generated by Tn5 transposase^21^. Peaks were called by MACS2 and HOMER was used for peak annotation^30,31^. After filtering, 7,755 peaks were detected at > 1 reads per million in at least one group.

The read counts were normalized by reference-adjusted reads per million (RRPM)^32^. After log2 and quantile transformation, the Limma test was used to select differential accessibility regions (DAR) between HIV-positive (n = 5) and HIV-negative (n = 4) participants in light of its high sensitivity for ATAC-seq analysis^33,34^. Motif enrichment analysis was performed using MEME suite SEA tool for ATAC-seq data to assess function of regions that showed different accessibility between the groups^35^.

ATAC-seq and RNA-seq data were integrated using xMWAS version 0.552, a data-driven integration tool that has been used for network visualization and analysis^36^. Partial least-squares (PLS) regression, a variable selection and dimensionality reduction method, was used to conduct pairwise association analysis between the 35 genes that were identified in RNA-seq data analysis as significant genes and 7755 peaks detected in ATAC-seq among all 9 subjects. If the association coefficiency between the two was ≥ 0.85, the gene transcripts and ATAC-seq peaks were considered correlated and connected with an edge to form a network. The network structure was analyzed by multilevel community detection method^37^.

## Results

### Participant demographics

There were nine participants included in this analysis, five with HIV and four without HIV. The median age of participants was 41 years (range 28–60), seven were female, and seven were cigarette smokers (four with HIV, 3 without HIV). For those with HIV, the median CD4 cell count was 966 cells/µl (range 728–1067).

### HIV alters the expression of sets of genes in alveolar macrophages

As shown in Table 1, gene set enrichment analysis of the RNA-seq data found six sets of genes altered in participants with HIV. The most upregulated gene set involved tumor necrosis factor-α (TNFα) signaling via nuclear factor (NF)-κB (normalized enrichment score of 2.63, permutation test *p* < 0.001), and four other gene sets showed a significant increase in expression: inflammatory response, hedgehog signaling, epithelial mesenchymal transition, angiogenesis (all with *p* ≤ 0.02). In contrast, only one gene set, fatty acid metabolism, showed a significant decrease in participants with HIV (− 1.49, *p* = 0.008).

**Table 1:** Gene set enrichment analysis (GSEA) of RNA-seq data from HIV + and HIV− patient alveolar macrophages.

Gene set	Normalized enrichment score	Nominal *P*-value	FDR *Q*-value	Upregulated group
TNFα-signaling via NF-kB	2.63	< 0.001	< 0.001	HIV +
Inflammatory response	2.14	< 0.001	< 0.001	HIV +
Hedgehog signaling	1.8	0.002	0.004	HIV +
Epithelial mesenchymal transition	1.64	< 0.001	0.024	HIV +
Angiogenesis	1.62	0.020	0.024	HIV+
Fatty acid metabolism	− 1.49	0.008	0.153	HIV−

Only gene sets with a nominal *p*-value < 0.01 or Benjamini–Hochberg FDR corrected *q*-value < 0.05 are presented.

### Gene expression is altered in alveolar macrophages from people with HIV

Table 2 presents RNA-seq data on significant differential expression of genes in alveolar macrophages between people with and without HIV. A total of 35 genes were identified, with functions ranging from inflammatory response to epithelial-mesenchymal transition. The greatest log-fold change was seen in A disintegrin and metalloproteinase with thrombospondin motifs 1 (ADAMTS1), which was downregulated in people with HIV (log fold change − 4.53, p < 0.001). The raw p-value of the top six genes were all under 0.001, with FDR corrected *Q* < 0.25. In the remaining genes, *p*-values were < 0.05 with FDR corrected *Q* values > 0.25.

**Table 2:** Genes with significant differential expression between HIV+ and HIV− (FDR *q*-value < 0.25) and their contribution to gene set enrichment (raw *p*-value < 0.05 by differential expression analysis).

Gene name	Log2 (fold change)	*P* value	FDR Q value	Gene set enrichment contributions
ADAMTS1	− 4.53	4.15E-07	0.01	Androgen response
TEP1	− 0.32	4.66E-06	0.04	N.A
ALDH6A1	− 0.60	1.56E-05	0.09	Oxidative phosphorylation/Heme metabolism
SLC2A14	4.46	5.31E-05	0.24	N.A
TUBB6	0.71	7.37E-05	0.24	N.A
KCNAB1	− 2.10	7.91E-05	0.24	N.A
IL6	2.23	0.007	> 0.25	TNFα/Inflammatory response/EMT
IL23A	2.00	0.009	> 0.25	TNFα
BMP2	2.10	0.022	> 0.25	TNFα
EFNA1	1.42	0.028	> 0.25	TNFα
F3	1.09	0.033	> 0.25	TNFα /Inflammatory response
PLAU	0.69	0.034	> 0.25	TNFα
LIF	1.65	0.043	> 0.25	TNFα /Inflammatory response
IL10	1.82	0.004	> 0.25	Inflammatory
CSF3	3.29	0.005	> 0.25	Inflammatory
PVR	0.64	0.018	> 0.25	Inflammatory/EMT
SLC4A4	3.64	0.019	> 0.25	Inflammatory
TNFSF15	1.30	0.025	> 0.25	Inflammatory
LCP2	0.38	0.035	> 0.25	Inflammatory
MET	1.94	0.042	> 0.25	Inflammatory
CXCL8	1.31	0.047	> 0.25	Inflammatory/EMT
PRRX1	2.10	0.002	> 0.25	EMT
DPYSL3	1.42	0.004	> 0.25	EMT
FBN2	2.37	0.020	> 0.25	EMT
ECM1	0.57	0.023	> 0.25	EMT
TIMP3	2.69	0.039	> 0.25	EMT
GREM1	1.63	0.041	> 0.25	EMT
COL4A2	0.68	0.042	> 0.25	EMT
THBD	2.01	0.003	> 0.25	Angiogenesis
TNFRSF21	1.19	0.005	> 0.25	Angiogenesis
LPL	0.85	0.038	> 0.25	Angiogenesis
CA2	− 1.13	0.002	> 0.25	Fatty acid metabolism
CD1D	− 1.13	0.010	> 0.25	Fatty acid metabolism
ACOT8	− 0.31	0.017	> 0.25	Fatty acid metabolism
ACSM3	− 0.87	0.024	> 0.25	Fatty acid metabolism

### HIV alters gene accessibility in alveolar macrophages

In Table 3, we present an analysis of the ATAC-seq data describing differentially accessible motif regions based on HIV status. These regions are paired with the relevant consensus sequences. Four motifs were differentially expressed between these two groups (*p* < 0.05). The full list of differentially accessible regions (DAR) according to HIV status is in Supplemental Table 1.

**Table 3:** Enriched sequence motifs in peak regions with different accesibility by ATAC-seq analysis.

Rank	Motif name	Consensus	*p*-value	*q*-value
1	VEZF1	GGRRRRGRRGGAGGGGGRGRRR	0.0008	0.318
2	ZNF8	TGTGGTATATCCATACAATGGA	0.0146	0.916
3	INSM1	TGTMAGGGGGCR	0.0169	0.916
4	ZBT17	SRRGGWGGGGGAGGGGMRR	0.0176	0.916

ATAC-seq data were then paired with RNA-seq data in Table 4 to allow a comparison between RNA expression levels and accessibility. In total, 115 genes demonstrated higher expression and higher accessibility, 67 with higher expression but lower accessibility, 52 with lower expression but greater accessibility, and 80 with lower expression and lower accessibility. A breakdown of the specific genes in each of these four categories can be found in Supplemental Table 2.

**Table 4:** Comparison of RNA-seq and ATAC-seq data for HIV-positive vs. HIV-negative alveolar macrophages.

	RNA expression higher	RNA expression lower
ATAC more available	115 (15,3)	52 (10,1)
ATAC less available	67 (4,0)	80 (8,2)

The number of genes with a more than 1.5-fold change in mRNA expression or chromatin accessiblity, as categorized by the mean differences of HIV-positive vs. HIV-negative samples, is in bold. Genes with a *p*-value < 0.05 in differential expression analysis by RNA-seq are represented by the first number in the parentheses and genes with a *p*-value < 0.05 in differentially accessible regions by ATAC-seq are represented by the second number in parentheses.

Details of gene names and peaks are listed in Supplemental Table 2.

### Distinct clusters of gene interactions are suggested by pairing RNA- and ATAC-seq data

**Figure 1:**
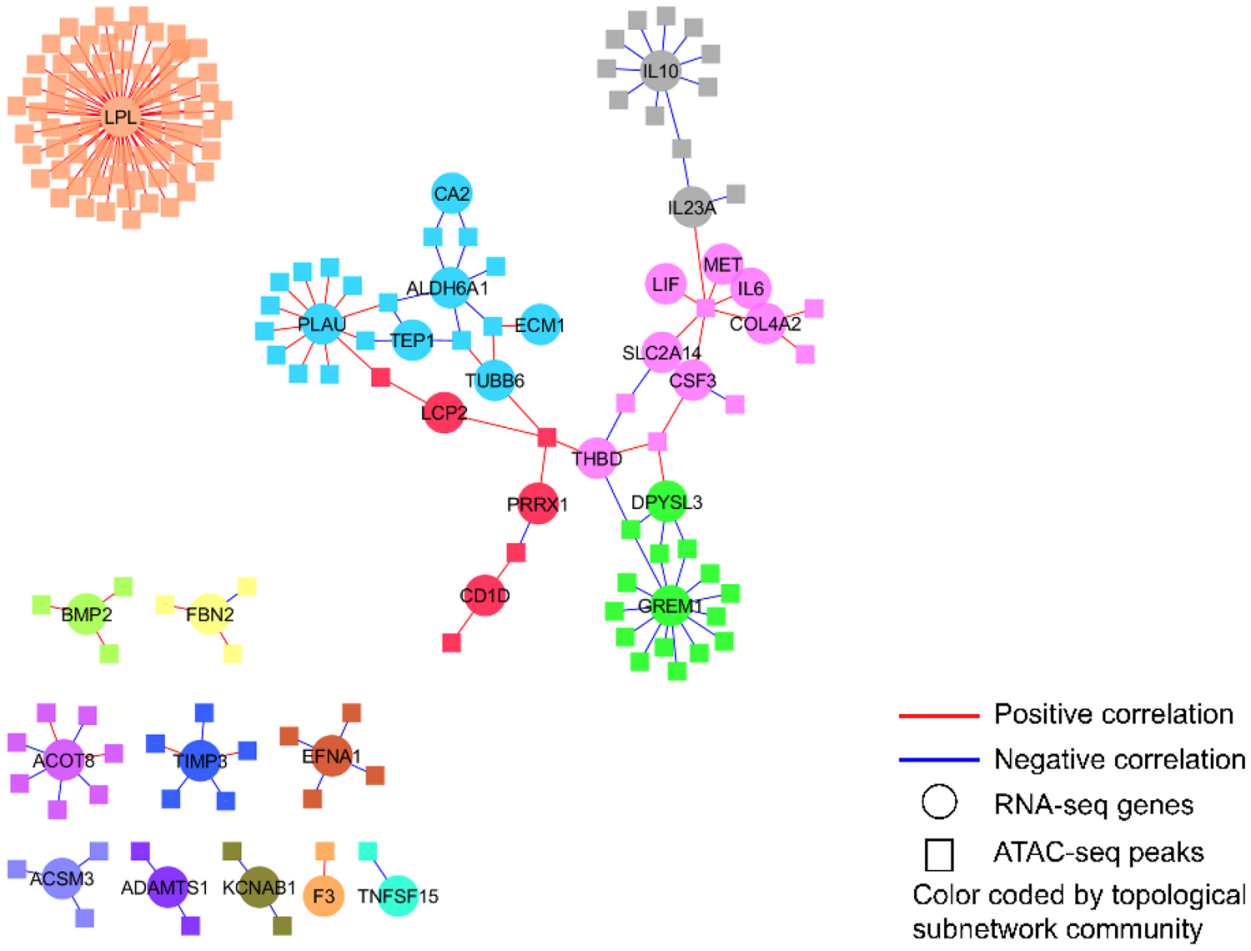
xMWAS data integration based on correlations between the RNA-seq (genes shown in green squares) and ATAC-seq data (peaks shown in orange squares). xMWAS data-driven integration identified three distinct clusters, one centered on lipoprotein lipase (LPL) with corresponding accesible peak regions from ATAC-seq and two others involving multiple genes from inflammatory pathways with shared accessibility regions forming an interconnected subnetwork structure. The nodes are color-coded by communites detected by mulilevel community detection algorithm that is based on topology of the network, while the edges represent the high correlations (|ρ|> 0.85, red: positive, blue: negative) between the connected two nodes.

To better understand the connection between gene expression and chromatin accessibility, xMWAS software ^36,38,39^, a data integration and differential network analysis tool to integrate the data of RNA-seq and ATAC-seq was used. Here, this approach was used to provide systems-level evaluation and visualization of how expression levels of significant genes were associated with the levels of chromatin region accessibility. Figure 1 shows the network of 35 significant genes and all correlated accessible peak regions. Topology-based community detection showed that lipoprotein lipase, an angiogenesis gene, forms a distinct subnetwork community with a number of accessible peak regions. In contrast, genes from inflammatory pathways demonstrate high associations with shared accessible regions and form an interconnected subnetwork structure. For example, a region (chr15: 70486203-70487082) was associated with several cytokines (IL 6, IL23A), MET (Proto-Oncogene, Receptor Tyrosine Kinase), LIF (Leukemia inhibitory factor), among others. This suggests that many of those genes are related in epigenetic regulation of their expression levels. Outside the LPL cluster and the interconnected subnetwork structure, 10 other genes formed small, distinct clusters, each associated with 1 to 6 peak regions.

## Discussion

In this pilot study, we used RNA-seq and ATAC-seq to compare gene expression and availability in alveolar macrophages of people with and without HIV. In doing so, we sought to determine whether pairing these complementary high-throughput assays could yield insights into the effects of HIV on macrophage phenotype and function. Despite the relatively small number of participants, we found multiple genes that differed in both expression level and accessibility, several of which have not previously been described as impacted by HIV, and many of which relate to key innate immune functions of the macrophage. Overall, we believe this study provides proof of concept for a useful analytic framework for investigating the pulmonary effects of HIV using high-throughput techniques. It further supports the transcriptome as a key locus of the virus’s effects on pulmonary innate immunity.

As the primary innate immune effector of the lung, macrophages are charged with monitoring the alveolar space for pathogenic threats, distinguishing friend from foe, and activating adaptive immunity should the need arise^10^. In addition, macrophages help control inflammation and promote wound healing after infectious or inflammatory insults^40^. Given their myriad important roles in the alveolar space, macrophage dysfunction has been linked to a broad spectrum of pulmonary diseases, including lung infections, emphysema, and pulmonary fibrosis^41–43^. In that regard, alveolar macrophages represent a novel and attractive therapeutic target, including for people with HIV who suffer from a disproportionate burden of lung disease^44^.

To better characterize macrophage dysfunction in HIV, we made use of two complementary high-throughput techniques, ATAC-seq and RNA-seq. The latter is a well-established means for assessing total levels of gene expression in response to various stimuli. These data can also be grouped by putative pathway to allow more global assessments that align with known biological functions^45^. ATAC-seq, in contrast, captures gene accessibility rather than total number of copies expressed^20^. In doing so, it generates a high-resolution map of nucleosome positions and transcription factor binding profiles. By providing a picture of which genes are ready and primed to be expressed, it offers transcriptomic and epigenetic data that are not available using gene expression levels alone^46^. By pairing these two techniques to compare alveolar macrophage phenotype in people with and without HIV, we sought to generate novel hypotheses and mechanisms of macrophage dysfunction in HIV.

The data generated by our analysis can be grouped in a number of illuminating ways. First, they can offer helpful support and confirmation for pathways known to be compromised in people with HIV. For example, TNFα signaling and inflammatory response genes both demonstrated significant trends toward upregulation in participants with HIV, a finding that accords well with prior work on macrophage inflammation and may be linked to changes in macrophage plasticity, which has been previously reported in HIV and is known to contribute to the development of viral reservoirs^47,48^. Next, these high-throughput data sets can be helpful in generating hypotheses for known clinical problems in HIV that lack a mechanism. For example, people with HIV are known to be at greater risk of COPD for unclear reasons, and the expression of ADAMTS1, which is involved in proteoglycan cleavage and vascular endothelial growth factor (VEGF) signaling and has been linked to COPD in other contexts, was significantly impaired in people with HIV (log fold change − 4.53, *p* < 0.001)^49^. Similarly, genes involved in epithelial-mesenchymal transition, a key process underlying extracellular matrix deposition, were upregulated by HIV, as was paired related homeobox 1 (PRRX1), a known promoter of fibrosis^50^. Taken together, these upregulated pathways and genes support the observation that people with HIV are at higher risk for lung fibrosis in clinical studies^51^.

Although the limited numbers in this pilot study do not allow for definitive conclusions, we did find that macrophages from people with and without HIV were distinguished by a number of differentially expressed genes, several of which have been previously implicated in HIV, including interleukin (IL)-6 and IL-10^52,53^. From a metholdologic standpoint, by visually integrating the RNA-seq and ATAC-seq datasets with xMWAS, we identified genes from inflammatory pathways as having multiple shared regions, forming an interconnected subnetwork structure. This analysis thus points us to potentially intervenable targets along the chain that may affect multiple downstream genes in parallel. In addition, we identified lipoprotein lipase as a key nexus in HIV-associated macrophage dysfunction, given its formation of a distinct cluster with accessible peak regions. In other contexts, lipoprotein lipase is known to regulate macrophage lipid accumulation, cellular metabolism, and phenotype^54^, but it has not yet been investigated in the context of the alveolar macrophage, nor with regards to HIV-associated pulmonary impairment, in particular.

Finally, our analysis points to a number of experimental approaches that may further enhance our understanding of HIV-mediated impairments in lung immunity. For example, we analyzed ATAC-seq data to look for patterns in accessibility among particular consensus sequences^55^. The identification of vascular endothelial zinc finger 1 (VEZF1) as a differententially accessible consensus sequence, for example, suggests a role for a future study with a DNA pull-down assay to determine which proteins may be interacting with the relevant sequence (and potential downstream functions that cells are primed for). Next, as shown in Table 4, we compared patterns of gene expression and gene accessibility and identified 119 genes with discordant expression and accessibility. Here, those with higher RNA expression and more availability (or lower expression and lower availability) may benefit from investigations of accessibility (e.g., histone acetylation). Those with opposite profiles (lower RNA expression and greater accessibility or greater expression and lower accessibility) could be investigated by assessing feedback loops to increase or limit production, respectively.

In addition to the limitations imposed by the small numbers of participants in the study, we recognize that our approach is subject to several other limitations. First and foremost, HIV is clearly not a static disease: its effects on the lung vary considerably between its early and later stages^56^. This study specifically examined the alveolar macrophages of subjects who were well-controlled on ART, and is unlikely to be generalizable to all subjects with HIV regardless of stage and immune status. We also recognize that the combination of two high-throughput assays do not offer a complete picture of the immune environment of the lung. An integrated-omics approach that assesses proteins, outside exposures, and the metabolome would provide further data on how HIV alters alveolar macrophage function. That said, we believe our approach gains substantial credability from our use of bronchscopically obtained alveolar macrophages from a human cohort, particularly given the known discordence between the lung microenvironment and systemic blood markers that has been noted in HIV^15^. Finally, we note that the small sample size makes it difficult to assess potential confounders (e.g., extent of immunodeficiency, tobacco use, ART regimen, etc.), which limits our ability to draw firm conclusions from the dataset. However, we believe the fact that our dataset accords with the existing literature in a number of key areas (e.g., TNFα, IL-6, IL-10) adds credibility to our overall approach.

In summary, in this proof-of-concept pilot study we paired RNA-seq and ATAC-seq analyses on alveolar macrophages from people with and without HIV, and found that this approach offers valuable data on key pathways and mechanisms that may underlie the increased risk of various pulmonary diseases in people with HIV. Further, we found clear advantages in pairing these two high-throughput techniques, as the data generated by each studies aids in the interpretation of the other. In addition, analysis of the paired data suggests potential follow-up studies that would be difficult to interpret with one assay in isolation. We believe an expansion of this approach to larger groups has the potential to generate strong hypotheses and suggest potential mechanisms that could lead to therapeutic options for alveolar macrophage dysfunction in people with HIV.

### Supplementary Information

Supplementary Information.

## Data Availability

Data from the study are available upon request from the corresponding author, Dr. Sara Auld (sauld@emory.edu).
