# Supplementary material for: Paired ATAC- and RNA-seq offer insight into the impact of HIV on alveolar macrophages: a pilot study
Source: Sci Rep. 2023 Sep 15;13:15276. doi: 10.1038/s41598-023-42644-7 (PMC10504379; doi:10.1038/s41598-023-42644-7)
Supplement: Supplementary file 1 — Supplementary Information. [file 41598_2023_42644_MOESM1_ESM.docx]

**SUPPLEMENT:**

**Paired ATAC- and RNA-seq offer insight into the impact of HIV on alveolar macrophages: a pilot study**

Bashar S. Staitieh^1,2^

Xin Hu^1^

Samantha M. Yeligar^1,3^

*Sara C. Auld^1,4^

^1^Division of Pulmonary, Allergy, Critical Care, and Sleep Medicine, Department of Medicine, School of Medicine, Emory University, Atlanta, Georgia

^2^Grady Health System, Atlanta, Georgia

^3^Veterans Affairs Atlanta Healthcare System, Decatur, Georgia

^4^Departments of Epidemiology and Global Health, Rollins School of Public Health, Emory University, Atlanta, Georgia


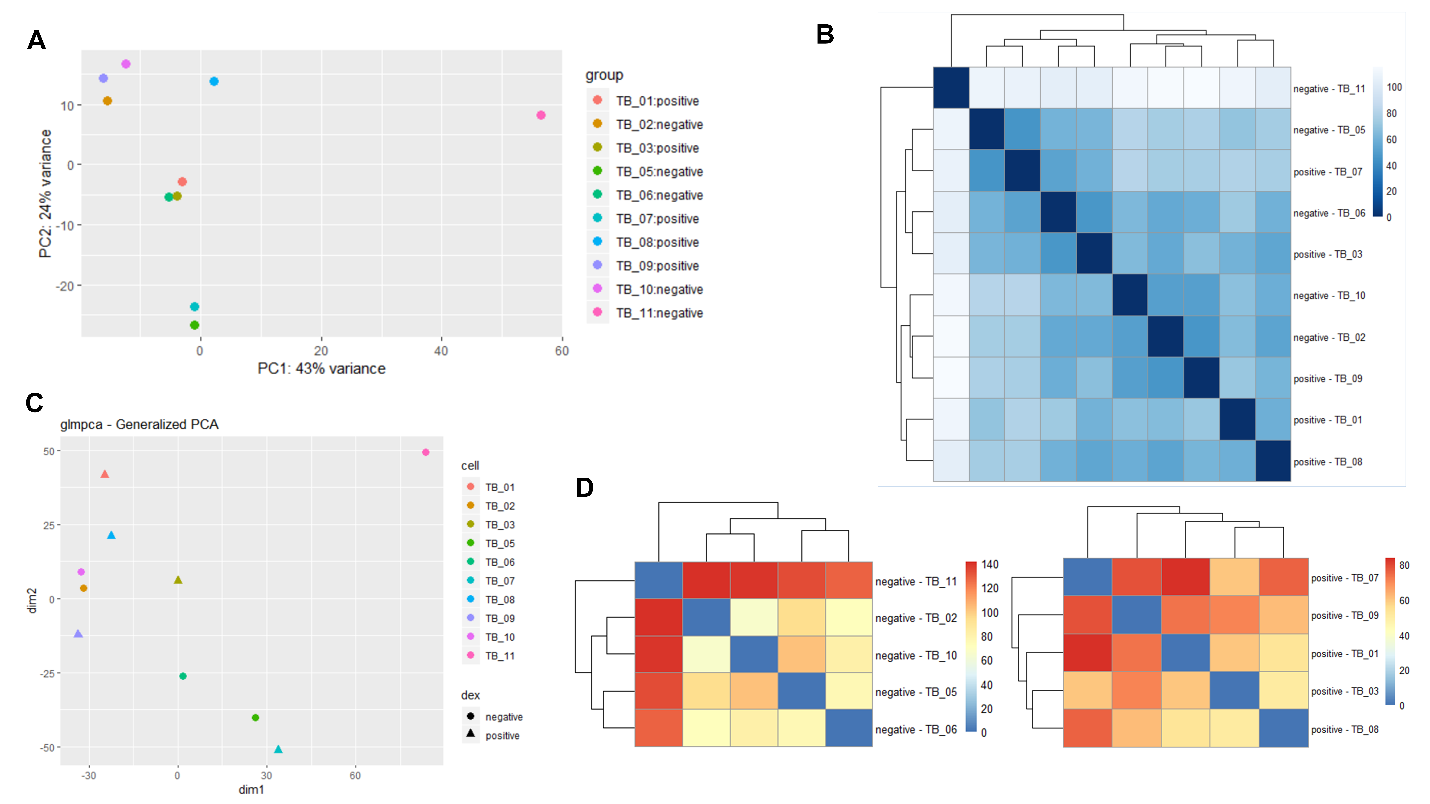


**Supplemental Figure 1.** Principle of component (PCA) analysis of normalized RNA-seq data (A) and unsupervised hierarchical clustering analysis (B) show that TB_11 was an outlier and exhibited greater difference to other samples than the general sample variation within the rest of samples. Raw RNA-seq counts was also examined using generalized version of principal components analysis (GLM-PCA) to show that TB_11 data was an outlier in raw data (C). Positive and negative samples were analyzed separately to show distance among samples, as shown in the scale bar in D, further confirming that TB_11 is much further to other samples than distances among the rest of the samples as an outlier.

**Supplemental Table 1.** List of differentially accessible regions (DAR) between alveolar macrophages from HIV-positive and HIV-negative participants (raw p-values < 0.05).

| **chr** | **start** | **end** | **strand** | **gene** | **Annotation** | **Detailed.Annotation** | **Distance.to.TSS** | **Entrez.ID** | **P.value** |
| --- | --- | --- | --- | --- | --- | --- | --- | --- | --- |
| chr13 | 42991882 | 42992867 | + | EPSTI1 | promoter-TSS (NM_001331228) | promoter-TSS (NM_001331228) | -62 | 94240 | 0.001 |
| chr5 | 69844085 | 69845195 | + | LOC653080 | promoter-TSS (NR_029426) | promoter-TSS (NR_029426) | -29 | 653080 | 0.003 |
| chr15 | 21656389 | 21657313 | + | OR4M2 | Intergenic | L1P4b\|LINE\|L1 | 18942 | 390538 | 0.003 |
| chr13 | 36999966 | 37001166 | + | EXOSC8 | promoter-TSS (NM_181503) | promoter-TSS (NM_181503) | 25 | 11340 | 0.003 |
| chr13 | 77329030 | 77330104 | + | MYCBP2 | Intergenic | CpG | -2525 | 23077 | 0.004 |
| chr8 | 8625676 | 8626221 | + | CLDN23 | Intergenic | MLT1H\|LTR\|ERVL-MaLR | -76208 | 137075 | 0.004 |
| chr11 | 1.12E+08 | 1.12E+08 | + | ALG9 | non-coding (NR_147984, exon 1 of 16) | non-coding (NR_147984, exon 1 of 16) | 117 | 79796 | 0.007 |
| chr4 | 56386994 | 56387893 | + | AASDH | promoter-TSS (NM_001286668) | promoter-TSS (NM_001286668) | 65 | 132949 | 0.007 |
| chr15 | 42736602 | 42737466 | + | CDAN1 | promoter-TSS (NM_138477) | promoter-TSS (NM_138477) | 94 | 146059 | 0.007 |
| chr5 | 65035308 | 65036313 | + | CWC27 | Intergenic | CpG | 266892 | 10283 | 0.007 |
| chrX | 17736874 | 17737697 | + | SCML1 | promoter-TSS (NM_006746) | promoter-TSS (NM_006746) | -164 | 6322 | 0.009 |
| chr10 | 1.14E+08 | 1.14E+08 | + | CCDC186 | promoter-TSS (NR_135815) | promoter-TSS (NR_135815) | -264 | 55088 | 0.009 |
| chr16 | 14974653 | 14975709 | + | PDXDC1 | 5' UTR (NM_015027, exon 1 of 23) | 5' UTR (NM_015027, exon 1 of 23) | 218 | 23042 | 0.010 |
| chr10 | 91923390 | 91924615 | + | BTAF1 | promoter-TSS (NM_003972) | promoter-TSS (NM_003972) | 23 | 9044 | 0.011 |
| chr9 | 1.05E+08 | 1.05E+08 | + | NIPSNAP3B | intron (NM_005502, intron 11 of 49) | L2\|LINE\|L2 | 71584 | 55335 | 0.011 |
| chr12 | 68807524 | 68808426 | + | MDM2 | promoter-TSS (NM_001145339) | promoter-TSS (NM_001145339) | -197 | 4193 | 0.013 |
| chr6 | 75601555 | 75602893 | + | SENP6 | 5' UTR (NM_001304792, exon 1 of 15) | 5' UTR (NM_001304792, exon 1 of 15) | 715 | 26054 | 0.014 |
| chr3 | 1.8E+08 | 1.8E+08 | + | USP13 | promoter-TSS (NM_003940) | promoter-TSS (NM_003940) | -5 | 8975 | 0.014 |
| chr5 | 34244279 | 34245374 | + | C1QTNF3 | promoter-TSS (NR_146599) | promoter-TSS (NR_146599) | -102 | 114899 | 0.014 |
| chr1 | 1.54E+08 | 1.54E+08 | + | C1orf43 | promoter-TSS (NM_014847) | promoter-TSS (NM_014847) | 140 | 25912 | 0.015 |
| chr15 | 89893575 | 89894576 | + | AP3S2 | non-coding (NR_023361, exon 1 of 7) | non-coding (NR_023361, exon 1 of 7) | 310 | 10239 | 0.016 |
| chr4 | 7042356 | 7043425 | + | CCDC96 | promoter-TSS (NM_152293) | promoter-TSS (NM_152293) | 111 | 257236 | 0.016 |
| chr1 | 1.17E+08 | 1.17E+08 | + | MAN1A2 | promoter-TSS (NM_006699) | promoter-TSS (NM_006699) | -357 | 10905 | 0.017 |
| chr12 | 1.27E+08 | 1.27E+08 | + | LINC02405 | Intergenic | (CCGAGGCCGA)n\|Simple_repeat\|Simple_repeat | -105778 | 1.02E+08 | 0.017 |
| chr10 | 1E+08 | 1E+08 | + | ERLIN1 | promoter-TSS (NR_144759) | promoter-TSS (NR_144759) | -62 | 10613 | 0.018 |
| chr8 | 1.25E+08 | 1.25E+08 | + | TATDN1 | promoter-TSS (NM_005005) | promoter-TSS (NM_005005) | 146 | 83940 | 0.018 |
| chr12 | 69239228 | 69240292 | + | CPSF6 | intron (NM_007007, intron 1 of 9) | CpG | 223 | 11052 | 0.018 |
| chr1 | 64965904 | 64966909 | + | JAK1 | 5' UTR (NM_001321855, exon 1 of 26) | 5' UTR (NM_001321855, exon 1 of 26) | 163 | 3716 | 0.018 |
| chr22 | 29310920 | 29311445 | + | GAS2L1 | intron (NM_001278730, intron 5 of 5) | intron (NM_001278730, intron 5 of 5) | 4187 | 10634 | 0.018 |
| chr1 | 1.49E+08 | 1.49E+08 | + | PDE4DIP | promoter-TSS (NM_001350520) | promoter-TSS (NM_001350520) | -23 | 9659 | 0.019 |
| chr4 | 1.22E+08 | 1.22E+08 | + | KIAA1109 | Intergenic | CpG | -18038 | 84162 | 0.020 |
| chr7 | 1.44E+08 | 1.44E+08 | + | ARHGEF35 | non-coding (NR_126022, exon 1 of 5) | non-coding (NR_126022, exon 1 of 5) | 116 | 445328 | 0.020 |
| chr5 | 1.78E+08 | 1.78E+08 | + | PHYKPL | non-coding (NR_103508, exon 1 of 10) | non-coding (NR_103508, exon 1 of 10) | 177 | 85007 | 0.020 |
| chr14 | 75069062 | 75069777 | + | ACYP1 | promoter-TSS (NR_126394) | promoter-TSS (NR_126394) | 64 | 97 | 0.020 |
| chr17 | 78186870 | 78187655 | + | AFMID | promoter-TSS (NM_003258) | promoter-TSS (NM_003258) | -55 | 125061 | 0.021 |
| chr2 | 10120209 | 10120879 | + | RRM2 | Intergenic | CpG | -2024 | 6241 | 0.021 |
| chr7 | 55571449 | 55572936 | + | VOPP1 | promoter-TSS (NM_001321249) | promoter-TSS (NM_001321249) | -30 | 81552 | 0.021 |
| chr6 | 96521472 | 96522631 | + | UFL1 | intron (NM_015323, intron 1 of 18) | CpG | 225 | 23376 | 0.021 |
| chr7 | 1.03E+08 | 1.03E+08 | + | RASA4B | intron (NM_001079877, intron 1 of 19) | intron (NM_001079877, intron 1 of 19) | 2924 | 1E+08 | 0.022 |
| chr12 | 1.12E+08 | 1.12E+08 | + | PTPN11 | promoter-TSS (NM_001320141) | promoter-TSS (NM_001320141) | 276 | 5781 | 0.022 |
| chr14 | 69152815 | 69153776 | + | DCAF5 | promoter-TSS (NM_001284208) | promoter-TSS (NM_001284208) | -98 | 8816 | 0.022 |
| chr7 | 1.13E+08 | 1.13E+08 | + | TMEM168 | promoter-TSS (NM_022484) | promoter-TSS (NM_022484) | 67 | 64418 | 0.022 |
| chr19 | 35748232 | 35748715 | + | LIN37 | TTS (NM_001281532) | TTS (NM_001281532) | 112 | 55957 | 0.023 |
| chrX | 1.56E+08 | 1.56E+08 | + | DDX11L16 | Intergenic | (TTAGGG)n\|Simple_repeat\|Simple_repeat | -2365 | 727856 | 0.023 |
| chr3 | 48663541 | 48664305 | + | LINC02585 | intron (NR_111921, intron 1 of 2) | CpG | 155 | 1.03E+08 | 0.023 |
| chr6 | 30742299 | 30743235 | + | FLOT1 | promoter-TSS (NM_001318875).2 | promoter-TSS (NM_001318875).2 | 84 | 10211 | 0.023 |
| chr6 | 52361549 | 52363021 | + | PAQR8 | intron (NM_133367, intron 1 of 1) | CpG | 157 | 85315 | 0.024 |
| chr12 | 31073566 | 31074465 | + | DDX11-AS1 | promoter-TSS (NR_038927) | promoter-TSS (NR_038927) | -168 | 1.01E+08 | 0.025 |
| chr18 | 3593608 | 3594602 | + | DLGAP1-AS1 | promoter-TSS (NR_024101) | promoter-TSS (NR_024101) | -9 | 649446 | 0.025 |
| chr10 | 87817864 | 87818963 | + | CFL1P1 | promoter-TSS (NR_028492) | promoter-TSS (NR_028492) | 100 | 142913 | 0.025 |
| chr2 | 1.12E+08 | 1.12E+08 | + | ZC3H8 | exon (NM_032494, exon 1 of 9) | exon (NM_032494, exon 1 of 9) | 159 | 84524 | 0.026 |
| chr19 | 2269242 | 2269825 | + | OAZ1 | promoter-TSS (NM_004152) | promoter-TSS (NM_004152) | 47 | 4946 | 0.026 |
| chr17 | 57987610 | 57988769 | + | VEZF1 | promoter-TSS (NM_007146) | promoter-TSS (NM_007146) | 65 | 7716 | 0.027 |
| chr11 | 65857961 | 65858484 | + | CFL1 | 5' UTR (NM_005507, exon 1 of 4) | 5' UTR (NM_005507, exon 1 of 4) | 111 | 1072 | 0.027 |
| chr11 | 1.02E+08 | 1.02E+08 | + | BIRC2 | promoter-TSS (NM_001256166) | promoter-TSS (NM_001256166) | -58 | 329 | 0.027 |
| chr20 | 33489610 | 33490697 | + | CBFA2T2 | promoter-TSS (NM_001032999) | promoter-TSS (NM_001032999) | 31 | 9139 | 0.028 |
| chr19 | 11155691 | 11156259 | + | SPC24 | promoter-TSS (NM_001317033) | promoter-TSS (NM_001317033) | -163 | 147841 | 0.028 |
| chr12 | 74537325 | 74538167 | + | ATXN7L3B | promoter-TSS (NM_001136262) | promoter-TSS (NM_001136262) | -25 | 552889 | 0.028 |
| chr10 | 45726698 | 45727852 | + | WASHC2C | promoter-TSS (NM_001169106) | promoter-TSS (NM_001169106) | 75 | 253725 | 0.028 |
| chr2 | 1.78E+08 | 1.78E+08 | + | TTC30B | promoter-TSS (NM_152517) | promoter-TSS (NM_152517) | -105 | 150737 | 0.029 |
| chr12 | 56359929 | 56360769 | + | STAT2 | promoter-TSS (NM_005419) | promoter-TSS (NM_005419) | -96 | 6773 | 0.029 |
| chr15 | 1E+08 | 1E+08 | + | SPATA41 | non-coding (NR_028139, exon 1 of 2) | non-coding (NR_028139, exon 1 of 2) | 180 | 388182 | 0.029 |
| chr20 | 53642432 | 53643478 | + | LOC105372672 | Intergenic | Intergenic | 34601 | 1.05E+08 | 0.029 |
| chr14 | 64540142 | 64540893 | + | HSPA2 | promoter-TSS (NR_110550) | promoter-TSS (NR_110550) | 49 | 3306 | 0.029 |
| chr1 | 22024923 | 22026228 | + | LINC00339 | promoter-TSS (NR_109760) | promoter-TSS (NR_109760) | 71 | 29092 | 0.029 |
| chr14 | 52729757 | 52730886 | + | STYX | 5' UTR (NM_145251, exon 1 of 11) | 5' UTR (NM_145251, exon 1 of 11) | 156 | 6815 | 0.029 |
| chr15 | 84747564 | 84749132 | + | ZNF592 | promoter-TSS (NM_014630) | promoter-TSS (NM_014630) | -239 | 9640 | 0.030 |
| chr3 | 32570035 | 32571311 | + | DYNC1LI1 | exon (NM_001329135, exon 1 of 11) | exon (NM_001329135, exon 1 of 11) | 201 | 51143 | 0.030 |
| chr17 | 61927218 | 61928297 | + | INTS2 | 5' UTR (NM_001351695, exon 1 of 25) | 5' UTR (NM_001351695, exon 1 of 25) | 275 | 57508 | 0.031 |
| chr1 | 89524119 | 89525449 | + | LRRC8B | promoter-TSS (NM_001134476) | promoter-TSS (NM_001134476) | -54 | 23507 | 0.031 |
| chr4 | 1.56E+08 | 1.56E+08 | + | CTSO | intron (NM_001334, intron 1 of 7).2 | intron (NM_001334, intron 1 of 7).2 | 317 | 1519 | 0.031 |
| chr10 | 89643805 | 89645331 | + | PANK1 | promoter-TSS (NM_138316) | promoter-TSS (NM_138316) | -677 | 53354 | 0.031 |
| chr1 | 1.48E+08 | 1.48E+08 | + | GPR89B | exon (NM_001350180, exon 1 of 15) | exon (NM_001350180, exon 1 of 15) | 184 | 51463 | 0.031 |
| chr11 | 2399921 | 2400964 | + | TSSC4 | promoter-TSS (NM_005706) | promoter-TSS (NM_005706) | -46 | 10078 | 0.031 |
| chr7 | 12210747 | 12211761 | + | TMEM106B | promoter-TSS (NM_001134232) | promoter-TSS (NM_001134232) | 32 | 54664 | 0.031 |
| chr15 | 64825596 | 64826083 | + | PIF1 | promoter-TSS (NM_001286499) | promoter-TSS (NM_001286499) | -171 | 80119 | 0.031 |
| chr3 | 49539675 | 49540416 | + | BSN-DT | Intergenic | CpG | 14321 | 1E+08 | 0.032 |
| chr15 | 74872907 | 74873572 | + | SCAMP2 | exon (NM_005697, exon 1 of 9) | exon (NM_005697, exon 1 of 9) | 140 | 10066 | 0.032 |
| chr7 | 1.49E+08 | 1.49E+08 | + | ZNF777 | Intergenic | CpG | 38286 | 27153 | 0.032 |
| chr14 | 49598494 | 49599342 | + | RPS29 | promoter-TSS (NM_001351375) | promoter-TSS (NM_001351375) | -208 | 6235 | 0.032 |
| chr3 | 19946567 | 19948009 | + | RAB5A | promoter-TSS (NM_001330688) | promoter-TSS (NM_001330688) | 208 | 5868 | 0.033 |
| chr7 | 1.49E+08 | 1.49E+08 | + | ZNF786 | Intergenic | CpG | 25151 | 136051 | 0.033 |
| chr6 | 341191 | 341678 | + | DUSP22 | intron (NM_020185, intron 4 of 7) | intron (NM_020185, intron 4 of 7) | 49377 | 56940 | 0.033 |
| chr15 | 97960021 | 97961041 | + | ARRDC4 | promoter-TSS (NM_183376) | promoter-TSS (NM_183376) | -172 | 91947 | 0.033 |
| chr11 | 615142 | 616307 | + | IRF7 | promoter-TSS (NM_004031).2 | promoter-TSS (NM_004031).2 | 4 | 3665 | 0.033 |
| chr20 | 58306673 | 58307253 | + | PPP4R1L | intron (NR_003505, intron 2 of 16) | MLT1H2\|LTR\|ERVL-MaLR | 2476 | 55370 | 0.033 |
| chr9 | 37800514 | 37801592 | + | DCAF10 | exon (NM_024345, exon 1 of 7) | exon (NM_024345, exon 1 of 7) | 267 | 79269 | 0.034 |
| chr17 | 44503022 | 44503848 | + | GPATCH8 | 5' UTR (NM_001304942, exon 1 of 7) | 5' UTR (NM_001304942, exon 1 of 7) | 167 | 23131 | 0.034 |
| chr3 | 1.86E+08 | 1.86E+08 | + | ETV5 | promoter-TSS (NM_004454) | promoter-TSS (NM_004454) | -143 | 2119 | 0.034 |
| chr4 | 48830708 | 48831653 | + | OCIAD1 | promoter-TSS (NM_001168254) | promoter-TSS (NM_001168254) | -47 | 54940 | 0.034 |
| chr14 | 24215600 | 24216248 | + | MDP1 | TTS (NM_006156) | TTS (NM_006156) | 146 | 145553 | 0.035 |
| chr5 | 14871278 | 14872950 | + | ANKH | promoter-TSS (NM_054027) | promoter-TSS (NM_054027) | -329 | 56172 | 0.035 |
| chr8 | 90000776 | 90001982 | + | DECR1 | promoter-TSS (NM_001330575) | promoter-TSS (NM_001330575) | 27 | 1666 | 0.035 |
| chr1 | 37690335 | 37690865 | + | C1orf109 | promoter-TSS (NM_001350770) | promoter-TSS (NM_001350770) | -5 | 54955 | 0.035 |
| chr19 | 45584209 | 45585265 | + | OPA3 | exon (NM_025136, exon 1 of 2) | exon (NM_025136, exon 1 of 2) | 127 | 80207 | 0.035 |
| chr6 | 1.12E+08 | 1.12E+08 | + | FAM229B | promoter-TSS (NM_001033564) | promoter-TSS (NM_001033564) | -167 | 619208 | 0.035 |
| chr1 | 2.36E+08 | 2.36E+08 | + | NID1 | Intergenic | LTR10A\|LTR\|ERV1 | -32126 | 4811 | 0.035 |
| chr22 | 28883014 | 28883805 | + | ZNRF3 | promoter-TSS (NM_001206998) | promoter-TSS (NM_001206998) | -358 | 84133 | 0.036 |
| chr1 | 1.98E+08 | 1.98E+08 | + | DENND1B | non-coding (NR_125340, exon 1 of 20) | non-coding (NR_125340, exon 1 of 20) | 320 | 163486 | 0.036 |
| chr22 | 31630156 | 31631316 | + | PISD | promoter-TSS (NM_014338) | promoter-TSS (NM_014338) | 88 | 23761 | 0.036 |
| chr9 | 26946788 | 26947731 | + | IFT74 | promoter-TSS (NM_001099222) | promoter-TSS (NM_001099222) | -41 | 80173 | 0.037 |
| chr1 | 630182 | 630535 | + | LOC101928626 | Intergenic | Intergenic | -1349 | 1.02E+08 | 0.037 |
| chr17 | 28041065 | 28041889 | + | NLK | Intergenic | A-rich\|Low_complexity\|Low_complexity | -1185 | 51701 | 0.037 |
| chr13 | 46295426 | 46296531 | + | LINC00563 | TTS (NR_047493) | TTS (NR_047493) | 1866 | 1.01E+08 | 0.037 |
| chr4 | 3292217 | 3293232 | + | RGS12 | Intergenic | CpG | -21423 | 6002 | 0.037 |
| chr13 | 50909520 | 50910821 | + | RNASEH2B | intron (NM_024570, intron 1 of 10) | CpG | 492 | 79621 | 0.038 |
| chr12 | 15881788 | 15882734 | + | STRAP | promoter-TSS (NM_007178) | promoter-TSS (NM_007178) | -93 | 11171 | 0.038 |
| chr6 | 26659262 | 26660347 | + | ZNF322 | promoter-TSS (NM_001242798) | promoter-TSS (NM_001242798) | -52 | 79692 | 0.038 |
| chr12 | 1.02E+08 | 1.02E+08 | + | DRAM1 | 5' UTR (NM_018370, exon 1 of 7) | 5' UTR (NM_018370, exon 1 of 7) | 221 | 55332 | 0.039 |
| chr5 | 32173795 | 32174712 | + | GOLPH3 | promoter-TSS (NM_022130) | promoter-TSS (NM_022130) | 66 | 64083 | 0.039 |
| chr12 | 6866841 | 6868375 | + | TPI1 | promoter-TSS (NM_001258026) | promoter-TSS (NM_001258026) | 79 | 7167 | 0.039 |
| chr10 | 43455302 | 43456111 | + | ZNF487 | intron (NM_001355444, intron 1 of 3) | CpG | 18595 | 642819 | 0.040 |
| chr17 | 6640541 | 6641515 | + | KIAA0753 | promoter-TSS (NM_014804) | promoter-TSS (NM_014804) | -101 | 9851 | 0.040 |
| chr17 | 63741274 | 63742825 | + | STRADA | promoter-TSS (NM_001165969) | promoter-TSS (NM_001165969) | -79 | 92335 | 0.040 |
| chr1 | 2.26E+08 | 2.26E+08 | + | ACBD3 | exon (NM_022735, exon 1 of 8) | exon (NM_022735, exon 1 of 8) | 131 | 64746 | 0.040 |
| chr17 | 8934982 | 8935728 | + | PIK3R5 | intron (NM_001142633, intron 1 of 18) | intron (NM_001142633, intron 1 of 18) | -22838 | 23533 | 0.040 |
| chr6 | 37432854 | 37433786 | + | CMTR1 | 5' UTR (NM_015050, exon 1 of 24) | 5' UTR (NM_015050, exon 1 of 24) | 189 | 23070 | 0.041 |
| chr5 | 71455407 | 71456496 | + | BDP1 | exon (NM_018429, exon 1 of 39).2 | exon (NM_018429, exon 1 of 39).2 | 336 | 55814 | 0.041 |
| chr1 | 1.46E+08 | 1.46E+08 | + | RNF115 | promoter-TSS (NM_006468) | promoter-TSS (NM_006468) | -7 | 27246 | 0.041 |
| chr7 | 1458856 | 1459936 | + | MICALL2 | promoter-TSS (NM_182924) | promoter-TSS (NM_182924) | 77 | 79778 | 0.041 |
| chr5 | 10010 | 11782 | + | PLEKHG4B | Intergenic | (TAACCC)n\|Simple_repeat\|Simple_repeat | -81255 | 153478 | 0.042 |
| chr1 | 1.67E+08 | 1.67E+08 | + | CD247 | intron (NM_198053, intron 1 of 7) | CpG | 63140 | 919 | 0.042 |
| chr17 | 37358788 | 37359714 | + | ACACA | promoter-TSS (NM_198836).2 | promoter-TSS (NM_198836).2 | -135 | 31 | 0.042 |
| chr19 | 18588501 | 18589649 | + | REX1BD | exon (NM_001100419, exon 2 of 5) | exon (NM_001100419, exon 2 of 5) | 390 | 55049 | 0.042 |
| chr13 | 79405603 | 79406718 | + | RBM26 | promoter-TSS (NM_022118) | promoter-TSS (NM_022118) | 61 | 64062 | 0.042 |
| chr12 | 12356809 | 12357850 | + | BORCS5 | promoter-TSS (NR_024061) | promoter-TSS (NR_024061) | 250 | 118426 | 0.043 |
| chr15 | 51621976 | 51623230 | + | DMXL2 | 5' UTR (NM_015263, exon 1 of 43) | 5' UTR (NM_015263, exon 1 of 43) | 167 | 23312 | 0.043 |
| chr14 | 34629621 | 34630583 | + | SNX6 | promoter-TSS (NM_021249) | promoter-TSS (NM_021249) | 81 | 58533 | 0.043 |
| chr9 | 178781 | 179370 | + | CBWD1 | promoter-TSS (NM_018491) | promoter-TSS (NM_018491) | 0 | 55871 | 0.044 |
| chr4 | 82373624 | 82374666 | + | HNRNPD | promoter-TSS (NM_002138) | promoter-TSS (NM_002138) | -149 | 3184 | 0.044 |
| chr3 | 1.02E+08 | 1.02E+08 | + | NFKBIZ | promoter-TSS (NM_031419) | promoter-TSS (NM_031419) | 100 | 64332 | 0.044 |
| chr3 | 42600143 | 42601390 | + | NKTR | 5' UTR (NM_001349125, exon 1 of 19) | 5' UTR (NM_001349125, exon 1 of 19) | 111 | 4820 | 0.045 |
| chr5 | 75236469 | 75237352 | + | ANKRD31 | promoter-TSS (NM_001164443) | promoter-TSS (NM_001164443) | -32 | 256006 | 0.045 |
| chr1 | 63593020 | 63594654 | + | PGM1 | promoter-TSS (NM_001172819) | promoter-TSS (NM_001172819) | 28 | 5236 | 0.045 |
| chr20 | 34363088 | 34363909 | + | ITCH | intron (NM_001324198, intron 1 of 24) | CpG | 263 | 83737 | 0.045 |
| chr9 | 83622773 | 83623679 | + | IDNK | intron (NR_046421, intron 1 of 3) | CpG | 177 | 414328 | 0.045 |
| chr4 | 36243786 | 36244963 | + | ARAP2 | promoter-TSS (NR_146893) | promoter-TSS (NR_146893) | -17 | 116984 | 0.045 |
| chr12 | 11170953 | 11171828 | + | SMIM10L1 | exon (NM_001271592, exon 1 of 1) | exon (NM_001271592, exon 1 of 1) | 209 | 1E+08 | 0.045 |
| chr14 | 75278450 | 75279210 | + | FOS | promoter-TSS (NM_005252) | promoter-TSS (NM_005252) | 52 | 2353 | 0.045 |
| chr22 | 23857176 | 23858054 | + | SLC2A11 | promoter-TSS (NM_001024939) | promoter-TSS (NM_001024939) | -238 | 66035 | 0.045 |
| chr19 | 51339614 | 51340519 | + | VSIG10L | exon (NM_001163922, exon 4 of 10) | exon (NM_001163922, exon 4 of 10) | 2058 | 147645 | 0.046 |
| chr12 | 64222039 | 64222738 | + | C12orf66 | promoter-TSS (NM_001300941) | promoter-TSS (NM_001300941) | -92 | 144577 | 0.046 |
| chr2 | 1.36E+08 | 1.36E+08 | + | CXCR4 | Intergenic | Intergenic | -120861 | 7852 | 0.046 |
| chr19 | 1269045 | 1270132 | + | CIRBP | intron (NR_023313, intron 1 of 5) | CpG-13015 | 320 | 1153 | 0.046 |
| chr11 | 57567633 | 57568541 | + | UBE2L6 | promoter-TSS (NM_004223) | promoter-TSS (NM_004223) | 243 | 9246 | 0.047 |
| chr3 | 1.39E+08 | 1.39E+08 | + | CEP70 | promoter-TSS (NM_001288966) | promoter-TSS (NM_001288966) | -203 | 80321 | 0.047 |
| chr17 | 28335255 | 28336044 | + | TNFAIP1 | promoter-TSS (NM_001267778) | promoter-TSS (NM_001267778) | 127 | 7126 | 0.047 |
| chr3 | 1.02E+08 | 1.02E+08 | + | ZBTB11 | promoter-TSS (NM_014415) | promoter-TSS (NM_014415) | -51 | 27107 | 0.047 |
| chr10 | 79347301 | 79348366 | + | PPIF | intron (NM_005729, intron 1 of 5) | CpG | 369 | 10105 | 0.049 |
| chr1 | 1.84E+08 | 1.84E+08 | + | NCF2 | promoter-TSS (NM_000433) | promoter-TSS (NM_000433) | 115 | 4688 | 0.049 |
| chr2 | 1.27E+08 | 1.27E+08 | + | GYPC | promoter-TSS (NM_016815) | promoter-TSS (NM_016815) | 99 | 2995 | 0.049 |
| chr6 | 33391193 | 33392041 | + | KIFC1 | promoter-TSS (NM_002263) | promoter-TSS (NM_002263) | 81 | 3833 | 0.050 |
| chr8 | 41828050 | 41829067 | + | ANK1 | intron (NM_001142446, intron 1 of 42) | CpG | -30936 | 286 | 0.050 |
| chr12 | 9758592 | 9759362 | + | CD69 | intron (NM_001781, intron 1 of 4) | AluY\|SINE\|Alu | 1924 | 969 | 0.050 |

**Supplemental Table 2.** Genes with more than 1.5-fold change in mRNA expression or chromatin accessibility indicated by annotated peak, categorized by the log_2_ fold change in mean differences of HIV-positive vs HIV-negative alveolar macrophages.

| **gene** | **RNA_fold.change.**  **log2** | **RNA_pvalue** | **ATAC_fold.change.**  **log2** | **ATAC_pvalue** | **peak** | **Detailed.Annotation** | **Distance.to.TSS** | **ATAC (HIV vs non-HIV)** | **RNA (HIV vs non-HIV)** |
| --- | --- | --- | --- | --- | --- | --- | --- | --- | --- |
| ABTB2 | 0.85 | 0.091 | 0.26 | 0.098 | chr11_34357250_34358221 | 5' UTR (NM_145804, exon 1 of 17) | 273 | + | + |
| APBB2 | 0.63 | >0.1 | 0.21 | >0.1 | chr4_41214298_41214854 | promoter-TSS (NM_173075) | 42 | + | + |
| APBB2 | 0.63 | >0.1 | 0.12 | >0.1 | chr4_40887609_40888254 | intron (NM_004307, intron 12 of 17) | -30739 | + | + |
| AQP3 | 0.90 | 0.087 | 0.03 | >0.1 | chr9_33446450_33447778 | intron (NM_004925, intron 1 of 5) | 519 | + | + |
| ARHGAP31-AS1 | 0.78 | >0.1 | 0.11 | >0.1 | chr3_119322511_119323653 | promoter-TSS (NR_046748) | -322 | + | + |
| C17orf99 | 0.93 | >0.1 | 0.26 | >0.1 | chr17_78140244_78140922 | CpG | -5770 | + | + |
| C1orf61 | 0.88 | >0.1 | 0.01 | >0.1 | chr1_156456469_156457580 | CpG | -27476 | + | + |
| C9orf43 | 0.72 | >0.1 | 0.19 | 0.089 | chr9_113410132_113410929 | promoter-TSS (NM_001278630) | -114 | + | + |
| CBWD3 | 0.66 | 0.049 | 0.20 | >0.1 | chr9_68241665_68242293 | promoter-TSS (NM_001291821) | 34 | + | + |
| CCL20 | 1.00 | >0.1 | 0.03 | >0.1 | chr2_227817960_227819109 | TTS (NM_004591) | 4692 | + | + |
| CCL3 | 1.11 | >0.1 | 0.23 | >0.1 | chr17_36089828_36090716 | promoter-TSS (NR_111964).3 | -112 | + | + |
| CCL4L2 | 1.59 | 0.076 | 0.11 | >0.1 | chr17_36210658_36211370 | promoter-TSS (NM_001291468).2 | -49 | + | + |
| CD80 | 1.08 | >0.1 | 0.34 | >0.1 | chr3_119562358_119563057 | Intergenic | -3073 | + | + |
| CHMP3 | 0.60 | 0.063 | 0.11 | >0.1 | chr2_86562673_86563707 | CpG | 307 | + | + |
| CHRNA1 | 0.86 | >0.1 | 0.07 | >0.1 | chr2_174771670_174772558 | Intergenic | -7642 | + | + |
| CLEC19A | 0.60 | >0.1 | 0.30 | >0.1 | chr16_19294097_19294834 | L1MEd\|LINE\|L1 | 8682 | + | + |
| CXCL2 | 1.00 | 0.053 | 0.29 | 0.080 | chr4_74098692_74099655 | 5' UTR (NM_002089, exon 1 of 4) | 107 | + | + |
| CXCL3 | 0.81 | >0.1 | 0.05 | >0.1 | chr4_74038283_74039279 | promoter-TSS (NM_002090) | -8 | + | + |
| CXCL8 | 1.31 | 0.047 | 0.40 | >0.1 | chr4_73743823_73744679 | TTS (NM_000584) | 3745 | + | + |
| CXCL8 | 1.31 | 0.047 | 0.28 | >0.1 | chr4_73740408_73741331 | intron (NM_001354840, intron 1 of 2) | 363 | + | + |
| DRD5 | 1.73 | >0.1 | 0.11 | >0.1 | chr4_9691300_9692058 | Intergenic | -89955 | + | + |
| DUSP22 | 0.14 | >0.1 | 0.60 | 0.033 | chr6_291665_293126 | non-coding (NR_104475, exon 1 of 4) | 338 | + | + |
| EFR3B | 0.64 | >0.1 | 0.11 | >0.1 | chr2_25041403_25042642 | promoter-TSS (NM_014971) | -82 | + | + |
| EGR1 | 0.88 | >0.1 | 0.06 | >0.1 | chr5_138464015_138465565 | promoter-TSS (NM_001964) | -702 | + | + |
| ETS2 | 0.82 | 0.096 | 0.24 | >0.1 | chr21_38831271_38832090 | Intergenic | 25849 | + | + |
| ETS2 | 0.82 | 0.096 | 0.07 | >0.1 | chr21_38805050_38806143 | promoter-TSS (NM_005239) | -235 | + | + |
| FAM72C | 1.00 | >0.1 | 0.56 | >0.1 | chr1_145094787_145096057 | promoter-TSS (NM_207418) | -561 | + | + |
| FER1L5 | 0.75 | >0.1 | 0.15 | >0.1 | chr2_96665048_96665692 | intron (NM_001293083, intron 14 of 52) | 22633 | + | + |
| FLNB | 0.59 | >0.1 | 0.20 | >0.1 | chr3_57959226_57960336 | Intergenic | -48619 | + | + |
| FRG2 | 1.90 | >0.1 | 0.19 | >0.1 | chr4_190021656_190022769 | CpG | 5045 | + | + |
| GPIHBP1 | 2.01 | >0.1 | 1.07 | 0.090 | chr8_143219512_143220183 | Intergenic | 6654 | + | + |
| GTF2H2C | 0.91 | >0.1 | 0.09 | >0.1 | chr5_69595545_69596676 | TTS (NR_033417) | 35865 | + | + |
| HCAR2 | 1.19 | 0.026 | 0.01 | >0.1 | chr12_122646557_122647230 | Intergenic | 56464 | + | + |
| HERC2P3 | 0.82 | 0.064 | 0.29 | 0.068 | chr15_20506118_20506695 | promoter-TSS (NR_036432) | -226 | + | + |
| HES1 | 1.04 | 0.057 | 0.20 | 0.068 | chr3_194134398_194135260 | CpG | -1313 | + | + |
| HIST1H2BJ | 0.92 | 0.016 | 0.13 | >0.1 | chr6_27132478_27133356 | promoter-TSS (NM_021064) | -121 | + | + |
| HS1BP3-IT1 | 0.61 | >0.1 | 0.25 | >0.1 | chr2_20617896_20618751 | 3' UTR (NM_022460, exon 7 of 7) | -25775 | + | + |
| IER3 | 0.77 | >0.1 | 0.40 | 0.094 | chr6_30744077_30744734 | TTS (NR_149095) | 145 | + | + |
| IER3 | 0.77 | >0.1 | 0.19 | >0.1 | chr6_30752240_30752879 | Intergenic | -8009 | + | + |
| IL1B | 1.16 | >0.1 | 0.25 | >0.1 | chr2_112839346_112839994 | Intergenic | -2891 | + | + |
| INHBA | 1.37 | 0.080 | 0.19 | >0.1 | chr7_41870051_41870867 | Intergenic | -167351 | + | + |
| INHBA | 1.37 | 0.080 | 0.05 | >0.1 | chr7_41881482_41882216 | Arthur1B\|DNA\|hAT-Tip100 | -178741 | + | + |
| IRAK2 | 0.70 | >0.1 | 0.36 | >0.1 | chr3_10173631_10174412 | intron (NM_001570, intron 1 of 12) | 9142 | + | + |
| ITGA1 | 1.04 | 0.044 | 0.07 | >0.1 | chr5_52799849_52800565 | 5' UTR (NM_015946, exon 2 of 3) | 11905 | + | + |
| LINC00346 | 0.81 | 0.029 | 0.42 | 0.067 | chr13_110870057_110870688 | promoter-TSS (NR_027701) | -64 | + | + |
| LINC00656 | 1.00 | >0.1 | 0.12 | >0.1 | chr20_23147845_23148638 | Intergenic | -15605 | + | + |
| LINC00885 | 0.90 | >0.1 | 0.15 | >0.1 | chr3_196169794_196170406 | Intergenic | 27464 | + | + |
| LINC01336 | 0.91 | >0.1 | 0.19 | >0.1 | chr5_75052494_75054152 | promoter-TSS (NR_126375) | -680 | + | + |
| LIPN | 0.99 | >0.1 | 0.02 | >0.1 | chr10_88759537_88760201 | Intergenic | -1537 | + | + |
| LOC100505921 | 0.80 | >0.1 | 0.04 | >0.1 | chr7_7968335_7969414 | promoter-TSS (NR_110018) | -122 | + | + |
| LOC101927787 | 1.19 | >0.1 | 0.19 | >0.1 | chr1_234656975_234657614 | intron (NR_125944, intron 3 of 3) | 11005 | + | + |
| LOC101928438 | 0.60 | >0.1 | 0.11 | >0.1 | chr9_99819313_99820347 | promoter-TSS (NR_109802) | 59 | + | + |
| LOC101928461 | 1.15 | >0.1 | 0.07 | >0.1 | chr6_136593792_136594364 | intron (NM_005923, intron 21 of 29) | -35036 | + | + |
| LOC101929512 | 1.15 | >0.1 | 0.25 | >0.1 | chr2_161281070_161281420 | Intergenic | -26602 | + | + |
| LOC646626 | 0.65 | >0.1 | 0.12 | >0.1 | chr1_85276165_85277046 | non-coding (NR_045484, exon 1 of 2) | 247 | + | + |
| LOC729732 | 1.10 | 0.009 | 0.08 | >0.1 | chr8_12600545_12601755 | CpG | 64461 | + | + |
| LUCAT1 | 0.74 | >0.1 | 0.12 | >0.1 | chr5_91162508_91163262 | intron (NM_032119, intron 89 of 89) | 151517 | + | + |
| MCM10 | 1.71 | 0.089 | 0.07 | >0.1 | chr10_13161118_13161695 | promoter-TSS (NM_018518) | -148 | + | + |
| MIR1303 | 1.97 | >0.1 | 0.02 | >0.1 | chr5_154682365_154683262 | (CTCCCCC)n\|Simple_repeat\|Simple_repeat | -2963 | + | + |
| MIR193A | 1.35 | 0.010 | 0.05 | >0.1 | chr17_31558724_31559691 | promoter-TSS (NR_029710) | -789 | + | + |
| MIR320A | 1.25 | >0.1 | 0.04 | >0.1 | chr8_22244388_22245470 | promoter-TSS (NM_001722) | 114 | + | + |
| MIR4257 | 0.61 | >0.1 | 0.01 | >0.1 | chr1_150561026_150562417 | TTS (NM_019032) | 9792 | + | + |
| MIR4734 | 0.95 | 0.036 | 0.14 | >0.1 | chr17_38700785_38701258 | Intergenic | 1310 | + | + |
| MIR4734 | 0.95 | 0.036 | 0.09 | >0.1 | chr17_38701373_38701905 | TTS (NR_039887) | 692 | + | + |
| MIR4754 | 3.01 | 0.045 | 0.09 | >0.1 | chr19_58386568_58387471 | promoter-TSS (NM_001009) | -161 | + | + |
| MOB4 | 1.17 | >0.1 | 0.04 | >0.1 | chr2_197515612_197516380 | promoter-TSS (NM_015387) | -47 | + | + |
| MSH5 | 1.20 | >0.1 | 0.29 | >0.1 | chr6_31739715_31740108 | promoter-TSS (NM_025259) | -37 | + | + |
| MTRNR2L1 | 0.90 | >0.1 | 0.19 | >0.1 | chr17_22880000_22880340 | ALR/Alpha\|Satellite\|centr | 357059 | + | + |
| MUC3A | 3.05 | 0.040 | 0.23 | >0.1 | chr7_100957975_100958941 | exon (NM_005960, exon 2 of 12) | 9038 | + | + |
| NID1 | 0.86 | >0.1 | 0.76 | 0.035 | chr1_236097068_236097547 | LTR10A\|LTR\|ERV1 | -32126 | + | + |
| P2RY6 | 0.66 | >0.1 | 0.10 | >0.1 | chr11_73288709_73289724 | intron (NM_001277208, intron 1 of 1) | 17074 | + | + |
| PAX8-AS1 | 0.69 | >0.1 | 0.36 | >0.1 | chr2_113240876_113241429 | TTS (NR_047570) | 4883 | + | + |
| PHLDB3 | 0.61 | >0.1 | 0.09 | >0.1 | chr19_43503723_43504864 | CpG-14620 | 540 | + | + |
| PID1 | 0.90 | >0.1 | 0.14 | >0.1 | chr2_229415990_229416791 | intron (NM_139072, intron 9 of 12) | -145049 | + | + |
| PKDCC | 1.53 | >0.1 | 0.19 | >0.1 | chr2_42101979_42102572 | Intergenic | 54254 | + | + |
| PKDCC | 1.53 | >0.1 | 0.03 | >0.1 | chr2_42098519_42099008 | Intergenic | 50742 | + | + |
| PLA2G4E-AS1 | 1.12 | >0.1 | 0.01 | >0.1 | chr15_41972374_41973317 | promoter-TSS (NR_120334) | 82 | + | + |
| PLLP | 0.72 | >0.1 | 0.06 | >0.1 | chr16_57299590_57300883 | AluJb\|SINE\|Alu | -15564 | + | + |
| PMFBP1 | 0.81 | >0.1 | 0.25 | >0.1 | chr16_72172236_72172795 | promoter-TSS (NM_001160213) | -65 | + | + |
| PMFBP1 | 0.81 | >0.1 | 0.16 | >0.1 | chr16_72202085_72202867 | FLAM_C\|SINE\|Alu | -30026 | + | + |
| RASA4B | 0.88 | >0.1 | 0.55 | 0.022 | chr7_102613564_102614100 | intron (NM_001079877, intron 1 of 19) | 2924 | + | + |
| RASD1 | 0.80 | >0.1 | 0.10 | >0.1 | chr17_17495883_17497161 | promoter-TSS (NM_016084) | -127 | + | + |
| RMRP | 0.99 | >0.1 | 0.07 | >0.1 | chr9_35657552_35658710 | promoter-TSS (NR_003051) | -113 | + | + |
| RN7SK | 0.91 | >0.1 | 0.05 | >0.1 | chr6_52995107_52996076 | promoter-TSS (NM_001512) | -29 | + | + |
| RNU11 | 0.76 | >0.1 | 0.09 | >0.1 | chr1_28648064_28649469 | TTS (NR_146731) | 166 | + | + |
| RNU4-2 | 1.17 | 0.077 | 0.03 | >0.1 | chr12_120291252_120292361 | promoter-TSS (NR_003137) | 97 | + | + |
| RNU5A-1 | 1.18 | >0.1 | 0.05 | >0.1 | chr15_65295758_65296748 | TTS (NR_002756) | 202 | + | + |
| RNU6ATAC | 0.84 | >0.1 | 0.05 | >0.1 | chr9_134164331_134165196 | promoter-TSS (NR_023344) | -199 | + | + |
| RNVU1-19 | 2.07 | >0.1 | 0.53 | >0.1 | chr1_120849976_120850997 | TTS (NR_104086) | 499 | + | + |
| RPPH1 | 1.19 | 0.096 | 0.28 | 0.070 | chr14_20342623_20343848 | promoter-TSS (NM_005484) | 176 | + | + |
| RSPH1 | 1.12 | >0.1 | 0.20 | >0.1 | chr21_42496055_42496742 | promoter-TSS (NM_001286506) | -44 | + | + |
| SEPT7P9 | 1.23 | >0.1 | 0.11 | >0.1 | chr10_38402496_38403564 | promoter-TSS (NR_027269) | -103 | + | + |
| SHF | 0.65 | >0.1 | 0.03 | >0.1 | chr15_45166563_45167676 | TTS (NM_138356) | 14088 | + | + |
| SLC1A2 | 1.55 | >0.1 | 0.12 | >0.1 | chr11_35303643_35304735 | intron (NM_001195728, intron 6 of 11) | 115369 | + | + |
| SLC41A2 | 0.61 | >0.1 | 0.02 | >0.1 | chr12_104958002_104958908 | promoter-TSS (NM_001352172) | -136 | + | + |
| SOCS2 | 0.84 | >0.1 | 0.23 | >0.1 | chr12_93570137_93571459 | promoter-TSS (NM_001270468) | -183 | + | + |
| SOCS3 | 0.71 | >0.1 | 0.29 | 0.082 | chr17_78337307_78337925 | Intergenic | 22463 | + | + |
| SOCS3 | 0.71 | >0.1 | 0.03 | >0.1 | chr17_78359367_78361012 | promoter-TSS (NM_003955) | -110 | + | + |
| SPRED1 | 0.64 | >0.1 | 0.13 | >0.1 | chr15_38251797_38252966 | promoter-TSS (NM_152594) | -470 | + | + |
| STAG3L5P | 0.85 | >0.1 | 0.04 | >0.1 | chr7_100335674_100336550 | promoter-TSS (NR_036569) | 33 | + | + |
| TAX1BP3 | 0.74 | >0.1 | 0.13 | >0.1 | chr17_3667793_3669188 | promoter-TSS (NM_031298) | 189 | + | + |
| TDRP | 0.74 | >0.1 | 0.38 | >0.1 | chr8_629314_631195 | intron (NM_001303100, intron 5 of 5) | -84473 | + | + |
| TEKT4P2 | 0.82 | >0.1 | 0.22 | >0.1 | chr21_9246222_9247214 | Intergenic | -116957 | + | + |
| TEKT4P2 | 0.82 | >0.1 | 0.11 | >0.1 | chr21_9125623_9126091 | MER57A1\|LTR\|ERV1 | 3904 | + | + |
| TEKT4P2 | 0.82 | >0.1 | 0.08 | >0.1 | chr21_9129398_9129986 | promoter-TSS (NR_038327) | 69 | + | + |
| THBD | 2.01 | 0.003 | 0.15 | >0.1 | chr20_23048504_23050075 | exon (NM_000361, exon 1 of 1) | 375 | + | + |
| TIFAB | 1.07 | >0.1 | 0.34 | >0.1 | chr5_135456147_135456939 | Intergenic | -4144 | + | + |
| TMEM163 | 0.60 | >0.1 | 0.53 | >0.1 | chr2_134739716_134740300 | Intergenic | -21007 | + | + |
| TMEM163 | 0.60 | >0.1 | 0.17 | >0.1 | chr2_134586318_134586965 | MLT1C\|LTR\|ERVL-MaLR | 132360 | + | + |
| TNFSF9 | 0.71 | >0.1 | 0.20 | >0.1 | chr19_6530507_6531587 | promoter-TSS (NM_003811) | 48 | + | + |
| TUBB6 | 0.71 | 0.000 | 0.16 | >0.1 | chr18_12307729_12309054 | CpG | 332 | + | + |
| VCAN | 1.09 | >0.1 | 0.22 | >0.1 | chr5_83306575_83307132 | intron (NM_022550, intron 7 of 7) | -164821 | + | + |
| VCAN | 1.09 | >0.1 | 0.03 | >0.1 | chr5_83473018_83474305 | intron (NM_004385, intron 1 of 14) | 1987 | + | + |
| WDR38 | 1.14 | >0.1 | 0.08 | >0.1 | chr9_124852855_124853627 | promoter-TSS (NM_001276376) | -176 | + | + |
| WDR63 | 0.63 | >0.1 | 0.05 | >0.1 | chr1_85061869_85062441 | promoter-TSS (NM_001288563) | -143 | + | + |
| ASB9P1 | -1.35 | >0.1 | 0.06 | >0.1 | chr15_92808962_92809742 | CpG | 13868 | + | - |
| BAHCC1 | -0.74 | 0.021 | 0.00 | >0.1 | chr17_81395160_81395545 | CpG | -4369 | + | - |
| CA2 | -1.13 | 0.002 | 0.06 | >0.1 | chr8_85463199_85464378 | promoter-TSS (NM_001293675) | -114 | + | - |
| CAND1.11 | -1.42 | >0.1 | 0.24 | 0.094 | chr11_10307522_10308295 | promoter-TSS (NR_103765) | -405 | + | - |
| CFAP43 | -0.96 | >0.1 | 0.02 | >0.1 | chr10_104232118_104232831 | promoter-TSS (NM_025145) | -97 | + | - |
| CLDN23 | -0.59 | 0.091 | 0.71 | 0.004 | chr8_8625676_8626221 | MLT1H\|LTR\|ERVL-MaLR | -76208 | + | - |
| COBLL1 | -0.74 | >0.1 | 0.01 | >0.1 | chr2_164841223_164842302 | promoter-TSS (NM_001278461) | -344 | + | - |
| CTLA4 | -1.06 | >0.1 | 0.05 | >0.1 | chr2_203811577_203812036 | Intergenic | -55982 | + | - |
| DACT3-AS1 | -0.62 | >0.1 | 0.36 | >0.1 | chr19_46660972_46661680 | promoter-TSS (NR_040041) | -152 | + | - |
| DBNDD1 | -1.00 | >0.1 | 0.43 | >0.1 | chr16_89994733_89996124 | non-coding (NR_003226, exon 11 of 11) | 14693 | + | - |
| DDIT4 | -0.96 | >0.1 | 0.10 | >0.1 | chr10_72297184_72298380 | CpG | 23863 | + | - |
| DDT | -1.81 | 0.080 | 0.09 | >0.1 | chr22_23974295_23974805 | promoter-TSS (NM_001084392) | -59 | + | - |
| DDX11L2 | -2.01 | 0.017 | 0.17 | >0.1 | chr2_113603524_113604448 | promoter-TSS (NR_024004) | -269 | + | - |
| DEGS2 | -1.04 | >0.1 | 0.09 | >0.1 | chr14_100192690_100193380 | CpG | -33360 | + | - |
| DNAH17-AS1 | -1.33 | 0.080 | 0.16 | >0.1 | chr17_78442498_78443324 | intron (NM_173628, intron 71 of 80) | -41999 | + | - |
| FAM86FP | -0.75 | >0.1 | 0.36 | >0.1 | chr12_8243090_8244075 | promoter-TSS (NR_024254) | -636 | + | - |
| FDXR | -0.73 | 0.039 | 0.24 | >0.1 | chr17_74872801_74873653 | promoter-TSS (NM_001258012) | -196 | + | - |
| FOXO6 | -0.68 | >0.1 | 0.13 | >0.1 | chr1_41360895_41361610 | promoter-TSS (NM_001291281) | -679 | + | - |
| GLIPR1L2 | -0.97 | 0.011 | 0.07 | >0.1 | chr12_75390720_75391528 | promoter-TSS (NM_001355030) | 54 | + | - |
| HKR1 | -0.25 | 0.088 | 0.96 | >0.1 | chr19_37317316_37318170 | promoter-TSS (NR_138105) | -139 | + | - |
| KIF5C | -0.47 | >0.1 | 0.60 | >0.1 | chr2_148881660_148881916 | intron (NM_004522, intron 1 of 25) | 6565 | + | - |
| LINC00324 | -1.01 | 0.016 | 0.01 | >0.1 | chr17_8221960_8223322 | non-coding (NR_026951, exon 2 of 3) | 1402 | + | - |
| LINC01168 | -2.30 | >0.1 | 0.01 | >0.1 | chr10_133009214_133009863 | Intergenic | 44004 | + | - |
| LINC01359 | -1.21 | >0.1 | 0.15 | >0.1 | chr1_65002205_65003367 | promoter-TSS (NR_119383) | -310 | + | - |
| LOC101927415 | -0.72 | >0.1 | 0.03 | >0.1 | chr12_123584035_123584776 | promoter-TSS (NR_110049) | -78 | + | - |
| LOC101927482 | -1.46 | >0.1 | 0.21 | >0.1 | chr2_196299809_196300278 | intron (NM_001348768, intron 13 of 28) | 40019 | + | - |
| LOC101930071 | -1.01 | >0.1 | 0.02 | >0.1 | chr19_42396308_42397300 | promoter-TSS (NR_126041) | -324 | + | - |
| LOC256880 | -0.72 | >0.1 | 0.10 | >0.1 | chr4_99950047_99950849 | promoter-TSS (NM_002106) | -46 | + | - |
| LYPD3 | -1.10 | 0.051 | 0.01 | >0.1 | chr19_43463190_43464382 | CpG-14618 | 1893 | + | - |
| MEP1A | -1.14 | >0.1 | 0.06 | >0.1 | chr6_46772624_46773234 | Intergenic | -20428 | + | - |
| MIR151B | -2.41 | 0.094 | 0.11 | >0.1 | chr14_100106362_100107075 | MER20B\|DNA\|hAT-Charlie | 2796 | + | - |
| MIR326 | -0.63 | >0.1 | 0.20 | >0.1 | chr11_75340607_75341185 | intron (NM_004041, intron 1 of 15) | -5710 | + | - |
| MIR3679 | -1.22 | >0.1 | 0.12 | >0.1 | chr2_134119601_134120206 | CpG | -7222 | + | - |
| MIR6069 | -0.66 | >0.1 | 0.02 | >0.1 | chr22_35352582_35353089 | Intergenic | -16036 | + | - |
| MMEL1 | -1.23 | >0.1 | 0.11 | >0.1 | chr1_2653620_2654357 | intron (NM_001242672, intron 4 of 6).2 | -20946 | + | - |
| MYLPF | -0.66 | >0.1 | 0.27 | >0.1 | chr16_30370294_30371339 | promoter-TSS (NM_015527) | -118 | + | - |
| NMUR1 | -1.52 | >0.1 | 0.08 | >0.1 | chr2_231545444_231545990 | Intergenic | -15246 | + | - |
| PFN1P2 | -0.66 | 0.099 | 0.07 | >0.1 | chr1_120529398_120530072 | intron (NR_144517, intron 21 of 41) | -95626 | + | - |
| PLTP | -0.61 | >0.1 | 0.11 | >0.1 | chr20_45910727_45911372 | promoter-TSS (NM_001242921) | -55 | + | - |
| PRKCZ | -0.59 | >0.1 | 0.20 | >0.1 | chr1_2133044_2133842 | CpG | 28727 | + | - |
| PRSS8 | -1.14 | 0.088 | 0.07 | >0.1 | chr16_31128771_31130057 | MIRb\|SINE\|MIR | 6348 | + | - |
| PRSS8 | -1.14 | 0.088 | 0.04 | >0.1 | chr16_31141871_31142806 | CpG | -6576 | + | - |
| RAB3C | -1.74 | 0.046 | 0.29 | 0.064 | chr5_58582668_58583305 | promoter-TSS (NM_138453) | -58 | + | - |
| RETN | -0.97 | 0.063 | 0.18 | >0.1 | chr19_7657013_7657665 | AluJr\|SINE\|Alu | -11747 | + | - |
| RNASET2 | -0.70 | 0.045 | 0.10 | >0.1 | chr6_166956378_166957598 | promoter-TSS (NM_003730) | -399 | + | - |
| SLC25A47 | -1.05 | >0.1 | 0.08 | >0.1 | chr14_100341244_100341918 | intron (NM_213645, intron 8 of 9) | 18244 | + | - |
| SPATA6 | -0.62 | 0.051 | 0.12 | >0.1 | chr1_48471863_48472551 | promoter-TSS (NM_001286239) | 1 | + | - |
| STEAP3-AS1 | -0.61 | >0.1 | 0.30 | >0.1 | chr2_119283841_119284430 | Intergenic | -35064 | + | - |
| TBX6 | -0.86 | 0.010 | 0.17 | >0.1 | chr16_30092516_30093069 | promoter-TSS (NM_004608) | -908 | + | - |
| TDRD3 | -1.65 | 0.003 | 0.00 | >0.1 | chr13_60396007_60396693 | promoter-TSS (NM_001146070) | -107 | + | - |
| TMEM18 | -0.10 | >0.1 | 0.78 | >0.1 | chr2_676446_677774 | promoter-TSS (NM_001352681) | 358 | + | - |
| WNT5B | -0.85 | >0.1 | 0.00 | >0.1 | chr12_1605319_1606367 | Intergenic | -11213 | + | - |
| ACTN2 | 0.59 | >0.1 | -0.07 | >0.1 | chr1_236713631_236715045 | intron (NM_001278344, intron 1 of 22) | 27884 | - | + |
| ANKRD26P1 | 0.21 | >0.1 | -0.59 | >0.1 | chr16_46387630_46388357 | Intergenic | 181104 | - | + |
| AXDND1 | 0.66 | >0.1 | -0.04 | >0.1 | chr1_179365519_179366429 | promoter-TSS (NR_073544) | 2 | - | + |
| BMP1 | 0.65 | >0.1 | -0.17 | >0.1 | chr8_22164452_22165613 | promoter-TSS (NM_001199) | -108 | - | + |
| C10orf111 | 1.14 | >0.1 | -0.15 | >0.1 | chr10_15096689_15097822 | promoter-TSS (NM_001265601) | 64 | - | + |
| CCL4L1 | 1.08 | >0.1 | -0.01 | >0.1 | chr17_36103396_36103961 | promoter-TSS (NR_111969) | 5 | - | + |
| CFAP61 | 1.06 | >0.1 | -0.18 | 0.077 | chr20_20052112_20052945 | promoter-TSS (NM_001278628) | 14 | - | + |
| CHRNA1 | 0.86 | >0.1 | -0.01 | >0.1 | chr2_174778424_174779157 | Intergenic | -14318 | - | + |
| CRB1 | 0.80 | >0.1 | -0.12 | >0.1 | chr1_197200888_197201782 | promoter-TSS (NR_135153) | -127 | - | + |
| DTL | 0.81 | >0.1 | -0.05 | >0.1 | chr1_212034815_212035904 | promoter-TSS (NM_001286230) | -194 | - | + |
| E2F7 | 0.62 | >0.1 | -0.30 | 0.058 | chr12_77065267_77066168 | promoter-TSS (NM_203394) | -137 | - | + |
| FAM184A | 0.72 | >0.1 | -0.10 | >0.1 | chr6_119146984_119147789 | intron (NM_001100411, intron 1 of 16) | 1807 | - | + |
| FRG1DP | 1.66 | >0.1 | -0.68 | >0.1 | chr20_28841821_28843383 | ALR/Alpha\|Satellite\|centr | -237788 | - | + |
| GUSBP3 | 1.17 | >0.1 | -0.30 | 0.056 | chr5_70585360_70586413 | promoter-TSS (NR_027386).2 | -84 | - | + |
| HIST1H2AI | 0.88 | >0.1 | -0.01 | >0.1 | chr6_27807639_27808551 | promoter-TSS (NM_003519) | -104 | - | + |
| HIST1H2AL | 0.62 | >0.1 | -0.17 | >0.1 | chr6_27864939_27865614 | promoter-TSS (NM_003511) | -53 | - | + |
| HIST1H4H | 0.63 | >0.1 | -0.18 | >0.1 | chr6_26285023_26286096 | promoter-TSS (NM_003543) | -60 | - | + |
| HIST4H4 | 0.60 | >0.1 | 0.00 | >0.1 | chr12_14770754_14771353 | promoter-TSS (NM_175054) | 78 | - | + |
| IL1RAP | 0.61 | >0.1 | -0.03 | >0.1 | chr3_190513366_190514292 | promoter-TSS (NM_001167931) | -222 | - | + |
| KCTD16 | 0.72 | >0.1 | -0.40 | >0.1 | chr5_144189723_144190417 | intron (NM_020768, intron 2 of 3) | 19197 | - | + |
| LOC101929512 | 1.15 | >0.1 | -0.14 | >0.1 | chr2_161278995_161280369 | Intergenic | -25039 | - | + |
| LOC441666 | 0.35 | >0.1 | -0.60 | >0.1 | chr10_41898795_41899017 | (AATGG)n\|Simple_repeat\|Simple_repeat | 469139 | - | + |
| LOC441666 | 0.35 | >0.1 | -0.60 | >0.1 | chr10_41904045_41904439 | (AATGG)n\|Simple_repeat\|Simple_repeat | 463803 | - | + |
| LOC441666 | 0.35 | >0.1 | -0.65 | >0.1 | chr10_41876680_41876860 | (ATGGA)n\|Simple_repeat\|Simple_repeat | 491275 | - | + |
| LOC441666 | 0.35 | >0.1 | -0.72 | >0.1 | chr10_41850694_41850952 | Intergenic | 517222 | - | + |
| LOC441666 | 0.35 | >0.1 | -0.75 | >0.1 | chr10_41903411_41903592 | (AATGG)n\|Simple_repeat\|Simple_repeat | 464544 | - | + |
| LOC441666 | 0.35 | >0.1 | -0.83 | >0.1 | chr10_41880879_41881076 | (ATGGA)n\|Simple_repeat\|Simple_repeat | 487068 | - | + |
| LOC441666 | 0.35 | >0.1 | -0.89 | 0.095 | chr10_42090680_42090946 | Intergenic | 277232 | - | + |
| LOC441666 | 0.35 | >0.1 | -0.92 | >0.1 | chr10_42089138_42089319 | Intergenic | 278817 | - | + |
| LOC645166 | 0.12 | >0.1 | -0.60 | >0.1 | chr1_143227775_143228047 | Intergenic | 194681 | - | + |
| LOC645166 | 0.12 | >0.1 | -0.61 | >0.1 | chr1_143254423_143255090 | Intergenic | 167836 | - | + |
| LOC645166 | 0.12 | >0.1 | -0.61 | >0.1 | chr1_143254225_143254317 | Intergenic | 168321 | - | + |
| LOC645166 | 0.12 | >0.1 | -0.62 | 0.086 | chr1_143243105_143243283 | Intergenic | 179398 | - | + |
| LOC645166 | 0.12 | >0.1 | -0.62 | 0.064 | chr1_143264005_143264325 | Intergenic | 158427 | - | + |
| LOC645166 | 0.12 | >0.1 | -0.63 | 0.084 | chr1_143262074_143263108 | (ATCGAATGGA)n\|Simple_repeat\|Simple_repeat | 160001 | - | + |
| LOC645166 | 0.12 | >0.1 | -0.68 | >0.1 | chr1_143212447_143213355 | Intergenic | 209691 | - | + |
| LRTM2 | 0.62 | >0.1 | -0.03 | >0.1 | chr12_1796007_1796656 | CpG | -23936 | - | + |
| LUCAT1 | 0.74 | >0.1 | -0.10 | >0.1 | chr5_91279699_91281002 | Intergenic | 34052 | - | + |
| MICAL2 | 0.64 | >0.1 | -0.09 | >0.1 | chr11_12110204_12111101 | promoter-TSS (NM_001346298) | 76 | - | + |
| MIR193A | 1.35 | 0.010 | -0.04 | >0.1 | chr17_31549238_31550532 | CpG | -10111 | - | + |
| MIR6883 | 1.63 | >0.1 | -0.02 | >0.1 | chr17_8138627_8139418 | (CCGGCT)n\|Simple_repeat\|Simple_repeat | 6049 | - | + |
| MRPS24 | 0.70 | >0.1 | -0.11 | >0.1 | chr7_43834598_43835223 | Intergenic | 34636 | - | + |
| MRPS24 | 0.70 | >0.1 | -0.16 | >0.1 | chr7_43869334_43870205 | promoter-TSS (NM_032014) | -223 | - | + |
| MTRNR2L1 | 0.90 | >0.1 | -0.04 | >0.1 | chr17_22977471_22977971 | ALR/Alpha\|Satellite\|centr | 454610 | - | + |
| MTRNR2L1 | 0.90 | >0.1 | -0.05 | >0.1 | chr17_22521236_22521599 | Intergenic | -1694 | - | + |
| MTRNR2L1 | 0.90 | >0.1 | -0.12 | >0.1 | chr17_24421139_24421620 | ALR/Alpha\|Satellite\|centr | 1898268 | - | + |
| MTRNR2L1 | 0.90 | >0.1 | -0.22 | >0.1 | chr17_23016137_23016575 | ALR/Alpha\|Satellite\|centr | 493245 | - | + |
| NAV2 | 0.79 | >0.1 | -0.08 | >0.1 | chr11_19712178_19713299 | promoter-TSS (NM_001244963) | -597 | - | + |
| RNU4ATAC | 0.65 | >0.1 | -0.09 | >0.1 | chr2_121530275_121531753 | TTS (NR_023343) | 134 | - | + |
| RNU5B-1 | 2.11 | >0.1 | -0.13 | >0.1 | chr15_65304247_65305259 | promoter-TSS (NR_002757) | 76 | - | + |
| SCLY | 0.67 | >0.1 | -0.04 | >0.1 | chr2_238060377_238061432 | promoter-TSS (NM_016510) | -20 | - | + |
| SHF | 0.65 | >0.1 | -0.03 | >0.1 | chr15_45200103_45200811 | tRNA-His-CAY_\|tRNA\|tRNA | 718 | - | + |
| SHF | 0.65 | >0.1 | -0.12 | >0.1 | chr15_45198353_45199167 | CpG | 2415 | - | + |
| SLC25A30-AS1 | 0.63 | >0.1 | -0.07 | >0.1 | chr13_45417670_45418824 | promoter-TSS (NR_047031) | 85 | - | + |
| SLC30A2 | 1.99 | >0.1 | -0.14 | >0.1 | chr1_26035247_26036301 | 3' UTR (NM_004455, exon 11 of 11) | 10364 | - | + |
| SNORD3D | 1.26 | >0.1 | -0.08 | >0.1 | chr17_19111678_19113147 | TTS (NR_006882) | 224 | - | + |
| TEC | 0.59 | 0.017 | -0.07 | >0.1 | chr4_48269166_48270393 | promoter-TSS (NM_003215) | 18 | - | + |
| TEKT4P2 | 0.82 | >0.1 | -0.29 | >0.1 | chr21_9324811_9325685 | CpG | -195487 | - | + |
| TEKT4P2 | 0.82 | >0.1 | -0.32 | >0.1 | chr21_9248545_9249047 | Intergenic | -119035 | - | + |
| TIMP4 | 0.68 | 0.008 | -0.22 | >0.1 | chr3_12193444_12193985 | Intergenic | -34363 | - | + |
| TNFRSF21 | 1.19 | 0.005 | -0.12 | >0.1 | chr6_47309253_47310132 | 5' UTR (NM_014452, exon 1 of 6) | 255 | - | + |
| TPBGL | 0.94 | 0.097 | -0.11 | >0.1 | chr11_75241181_75242109 | exon (NM_001195528, exon 1 of 1) | 740 | - | + |
| TUBA4A | 0.90 | 0.065 | -0.02 | >0.1 | chr2_219253700_219255191 | promoter-TSS (NM_006000) | 163 | - | + |
| UBE2MP1 | 0.12 | >0.1 | -0.60 | >0.1 | chr16_34586456_34586660 | Intergenic | 583833 | - | + |
| VCAN | 1.09 | >0.1 | -0.10 | >0.1 | chr5_83368311_83369323 | Intergenic | -102857 | - | + |
| WDR11-AS1 | 0.83 | >0.1 | -0.06 | >0.1 | chr10_120850995_120851788 | promoter-TSS (NR_033850) | -212 | - | + |
| XCR1 | 1.20 | >0.1 | 0.00 | >0.1 | chr3_46089134_46090114 | Intergenic | -62137 | - | + |
| ACTN1-AS1 | -0.59 | >0.1 | -0.12 | >0.1 | chr14_69042655_69043428 | Intergenic | 63359 | - | - |
| AKNA | -0.64 | 0.006 | -0.25 | 0.083 | chr9_114397657_114398822 | CpG | -3834 | - | - |
| ASB9P1 | -1.35 | >0.1 | -0.01 | >0.1 | chr15_92803770_92804563 | Intergenic | 8682 | - | - |
| ATP6V0E2-AS1 | -0.60 | >0.1 | -0.12 | >0.1 | chr7_149873350_149874197 | promoter-TSS (NR_027040) | 89 | - | - |
| BCL2L14 | -1.33 | >0.1 | -0.15 | >0.1 | chr12_12010092_12010940 | Intergenic | -60428 | - | - |
| C19orf67 | -0.66 | >0.1 | -0.06 | >0.1 | chr19_14081179_14081912 | TTS (NM_001277378) | 4250 | - | - |
| C1QTNF3 | -0.61 | >0.1 | -0.34 | 0.014 | chr5_34244279_34245374 | promoter-TSS (NR_146599) | -102 | - | - |
| C1QTNF6 | -1.17 | >0.1 | 0.00 | >0.1 | chr22_37198947_37199783 | CpG | -11075 | - | - |
| CALML6 | -1.60 | 0.067 | -0.13 | >0.1 | chr1_1908553_1909731 | CpG | -5685 | - | - |
| CAPN5 | -0.69 | >0.1 | -0.01 | >0.1 | chr11_77066309_77067153 | promoter-TSS (NM_004055) | -214 | - | - |
| CELA2A | -1.07 | >0.1 | -0.22 | >0.1 | chr1_15464197_15465449 | MIR3\|SINE\|MIR | 8095 | - | - |
| CHAC1 | -1.06 | >0.1 | -0.04 | >0.1 | chr15_40952903_40953765 | promoter-TSS (NM_024111) | -137 | - | - |
| CLDN23 | -0.59 | 0.091 | -0.05 | >0.1 | chr8_8701431_8702559 | promoter-TSS (NM_194284) | -161 | - | - |
| CP | -1.49 | >0.1 | -0.16 | >0.1 | chr3_149224825_149225615 | Intergenic | -3175 | - | - |
| CTH | -0.80 | 0.097 | -0.15 | >0.1 | chr1_70410621_70411527 | promoter-TSS (NM_001902) | -144 | - | - |
| CTNNA3 | -0.95 | >0.1 | -0.09 | >0.1 | chr10_67764000_67764748 | CpG | -68183 | - | - |
| DDIT4 | -0.96 | >0.1 | -0.02 | >0.1 | chr10_72273574_72274337 | promoter-TSS (NM_019058) | 36 | - | - |
| DEPTOR | -0.61 | >0.1 | -0.12 | >0.1 | chr8_119873446_119874335 | exon (NM_022783, exon 1 of 9) | 235 | - | - |
| DERL3 | -0.92 | >0.1 | 0.00 | >0.1 | chr22_23838831_23839563 | promoter-TSS (NM_198440) | -185 | - | - |
| EMBP1 | -0.38 | >0.1 | -0.64 | >0.1 | chr1_125176940_125177118 | Intergenic | 3657917 | - | - |
| EMBP1 | -0.38 | >0.1 | -0.64 | >0.1 | chr1_125169990_125170245 | Intergenic | 3651005 | - | - |
| EMBP1 | -0.38 | >0.1 | -0.82 | >0.1 | chr1_125183450_125184220 | Intergenic | 3664723 | - | - |
| FALEC | -0.87 | >0.1 | -0.13 | >0.1 | chr1_150515205_150516137 | promoter-TSS (NR_051960) | -86 | - | - |
| FAM117B | -0.64 | >0.1 | -0.01 | >0.1 | chr2_202634592_202635390 | promoter-TSS (NM_173511) | -187 | - | - |
| FAM53B-AS1 | -1.17 | >0.1 | -0.10 | >0.1 | chr10_124718076_124718599 | intron (NM_014661, intron 1 of 4) | 14309 | - | - |
| FAM53B-AS1 | -1.17 | >0.1 | -0.25 | 0.058 | chr10_124640713_124641652 | intron (NM_014661, intron 4 of 4) | -62846 | - | - |
| FLJ31356 | -1.50 | >0.1 | -0.05 | >0.1 | chr2_28406562_28407424 | intron (NM_005253, intron 2 of 3) | -12321 | - | - |
| GPT2 | -0.75 | 0.009 | 0.00 | >0.1 | chr16_46883675_46884534 | promoter-TSS (NM_133443) | -276 | - | - |
| GRB7 | -0.92 | >0.1 | -0.02 | >0.1 | chr17_39753723_39754897 | CpG | 14343 | - | - |
| GRK7 | -1.90 | 0.078 | -0.34 | >0.1 | chr3_141796548_141797856 | intron (NM_139209, intron 2 of 3) | 19001 | - | - |
| HHAT | -0.77 | >0.1 | -0.15 | >0.1 | chr1_210328734_210329597 | promoter-TSS (NM_001170580) | -131 | - | - |
| HIST1H2BE | -0.95 | >0.1 | -0.08 | >0.1 | chr6_26171664_26173054 | AluSc8\|SINE\|Alu | -11437 | - | - |
| HIST1H2BI | -1.42 | >0.1 | -0.19 | >0.1 | chr6_26272483_26273393 | promoter-TSS (NM_003525) | -38 | - | - |
| ITPKB-IT1 | -1.76 | 0.035 | -0.12 | >0.1 | chr1_226662733_226663398 | intron (NR_103784, intron 1 of 1) | 12003 | - | - |
| KLLN | -0.67 | >0.1 | -0.21 | >0.1 | chr10_87861638_87862739 | exon (NM_001126049, exon 1 of 1) | 1249 | - | - |
| LDB3 | -0.69 | >0.1 | -0.16 | >0.1 | chr10_86710987_86712607 | CpG | 43128 | - | - |
| LINC00638 | -0.99 | 0.079 | -0.04 | >0.1 | chr14_104815300_104816562 | CpG | -5270 | - | - |
| LINC00707 | -0.62 | >0.1 | -0.27 | >0.1 | chr10_6927064_6928378 | Intergenic | 148123 | - | - |
| LINC00881 | -0.96 | 0.061 | -0.03 | >0.1 | chr3_157088600_157089628 | promoter-TSS (NR_034008) | -767 | - | - |
| LINC00910 | -0.45 | >0.1 | -1.15 | >0.1 | chr17_43360353_43361679 | CpG | 27882 | - | - |
| LINC01011 | -0.85 | 0.062 | -0.08 | >0.1 | chr6_2988405_2990155 | CpG | 1313 | - | - |
| LOC100505942 | -0.60 | >0.1 | -0.01 | >0.1 | chr16_67521040_67521777 | intron (NR_104656, intron 1 of 1) | 7337 | - | - |
| LOC100506368 | -1.00 | >0.1 | -0.21 | >0.1 | chr11_86955092_86956330 | promoter-TSS (NR_038905) | 90 | - | - |
| LOC101927482 | -1.46 | >0.1 | -0.04 | >0.1 | chr2_196242102_196242784 | intron (NM_001348768, intron 19 of 28) | -17581 | - | - |
| LOC285819 | -1.29 | >0.1 | -0.35 | 0.061 | chr6_26473722_26474389 | intron (NR_038992, intron 3 of 3) | 8454 | - | - |
| LOC441081 | -0.61 | >0.1 | -0.32 | >0.1 | chr5_70450654_70451820 | TTS (NR_033417).2 | -35264 | - | - |
| LOC730101 | -0.98 | >0.1 | -0.01 | >0.1 | chr6_52664117_52664700 | promoter-TSS (NR_024405) | 7 | - | - |
| LRMP | -0.64 | >0.1 | -0.26 | >0.1 | chr12_25051835_25052896 | TTS (NM_001321724) | 118 | - | - |
| MIF-AS1 | -1.01 | >0.1 | 0.00 | >0.1 | chr22_23913691_23914438 | CpG | -15134 | - | - |
| MIR3124 | -0.90 | >0.1 | -0.04 | >0.1 | chr1_248825864_248826842 | promoter-TSS (NR_036070) | -24 | - | - |
| MIR4519 | -1.09 | >0.1 | -0.13 | >0.1 | chr16_30874767_30875738 | promoter-TSS (NR_039744) | 71 | - | - |
| MOG | -0.70 | >0.1 | -0.11 | >0.1 | chr6_29649634_29650373 | CpG | -6978 | - | - |
| NSUN5P2 | -0.70 | >0.1 | -0.03 | >0.1 | chr7_72924839_72925616 | CpG | 29536 | - | - |
| NUDT16P1 | -0.62 | 0.004 | -0.31 | >0.1 | chr3_131361108_131362387 | promoter-TSS (NR_002949) | -98 | - | - |
| ORM2 | -3.33 | 0.092 | -0.08 | >0.1 | chr9_114348831_114349387 | intron (NM_001317952, intron 14 of 20) | 19320 | - | - |
| PDXDC1 | -0.07 | >0.1 | -0.60 | 0.010 | chr16_14974653_14975709 | 5' UTR (NM_015027, exon 1 of 23) | 218 | - | - |
| PROC | -1.21 | >0.1 | -0.12 | >0.1 | chr2_127387077_127388397 | CpG | -30683 | - | - |
| PSAT1 | -2.46 | 0.011 | -0.36 | >0.1 | chr9_78296792_78297668 | exon (NM_058179, exon 1 of 9) | 155 | - | - |
| PTPN13 | -0.76 | >0.1 | -0.05 | >0.1 | chr4_86593976_86595322 | 5' UTR (NM_006264, exon 1 of 47) | 334 | - | - |
| RNASE2 | -0.99 | >0.1 | -0.04 | >0.1 | chr14_20970838_20971919 | Intergenic | 15907 | - | - |
| RNASET2 | -0.70 | 0.045 | -0.09 | >0.1 | chr6_166950919_166951629 | LTR13\|LTR\|ERVK | 5315 | - | - |
| RNVU1-7 | -0.76 | >0.1 | -0.16 | >0.1 | chr1_148038196_148039575 | promoter-TSS (NR_004426) | 31 | - | - |
| RPL34-AS1 | -0.86 | >0.1 | -0.11 | >0.1 | chr4_108619891_108620811 | promoter-TSS (NM_001319232) | 106 | - | - |
| RTCA-AS1 | -0.71 | >0.1 | -0.08 | >0.1 | chr1_100265868_100266540 | promoter-TSS (NM_003729) | -30 | - | - |
| SESN3 | -0.70 | >0.1 | -0.11 | >0.1 | chr11_95230897_95231778 | promoter-TSS (NM_144665) | -255 | - | - |
| SLA | -0.67 | >0.1 | -0.15 | >0.1 | chr8_133138992_133139911 | Intergenic | -36385 | - | - |
| SLC6A9 | -1.79 | >0.1 | -0.10 | >0.1 | chr1_44029815_44030606 | CpG | 913 | - | - |
| SMIM2-AS1 | -0.71 | 0.037 | -0.05 | >0.1 | chr13_44141671_44142584 | promoter-TSS (NR_104064) | -201 | - | - |
| ST20 | -0.63 | >0.1 | -0.07 | >0.1 | chr15_79923130_79924217 | promoter-TSS (NR_037652) | 81 | - | - |
| TEX41 | -1.81 | >0.1 | -0.12 | >0.1 | chr2_144660785_144661507 | Intergenic | -6821 | - | - |
| TGM3 | -2.03 | >0.1 | -0.05 | >0.1 | chr20_2301160_2301758 | MER112\|DNA\|hAT-Charlie | 5492 | - | - |
| TMEM9 | -0.64 | >0.1 | -0.02 | >0.1 | chr1_201170577_201171640 | CpG | 474 | - | - |
| TMEM9B-AS1 | -0.68 | 0.061 | -0.03 | >0.1 | chr11_8964114_8965278 | promoter-TSS (NR_073431) | 21 | - | - |
| TRAF3IP2-AS1 | -0.66 | 0.043 | -0.06 | >0.1 | chr6_111482147_111483228 | promoter-TSS (NR_034111) | -785 | - | - |
| UBE2Q2P2 | -0.77 | 0.075 | -0.21 | >0.1 | chr15_82354652_82355354 | promoter-TSS (NR_004847) | -133 | - | - |
| UCA1 | -1.00 | >0.1 | -0.21 | >0.1 | chr19_15836561_15837627 | TTS (NR_015379) | 8147 | - | - |
| UXT-AS1 | -0.69 | >0.1 | -0.06 | >0.1 | chrX_47658577_47659402 | non-coding (NR_045559, exon 1 of 5) | 156 | - | - |
| WHAMMP3 | -0.64 | 0.098 | -0.23 | >0.1 | chr15_22664324_22665411 | non-coding (NR_003521, exon 1 of 11) | 128 | - | - |
| ZBTB32 | -0.84 | >0.1 | -0.03 | >0.1 | chr19_35702235_35702922 | CpG | -1909 | - | - |
| ZNF718 | -0.63 | >0.1 | -0.27 | >0.1 | chr4_124233_125164 | 5' UTR (NM_001289930, exon 1 of 4) | 222 | - | - |
